# An epigenomic landscape of cervical intraepithelial neoplasia and cervical cancer using single‐base resolution methylome and hydroxymethylome

**DOI:** 10.1002/ctm2.498

**Published:** 2021-07-19

**Authors:** Yingxin Han, Liyan Ji, Yanfang Guan, Mengya Ma, Pansong Li, Yinge Xue, Yinxin Zhang, Wanqiu Huang, Yuhua Gong, Li Jiang, Xipeng Wang, Hong Xie, Boping Zhou, Jiayin Wang, Junwen Wang, Jinghua Han, Yuliang Deng, Xin Yi, Fei Gao, Jian Huang

**Affiliations:** ^1^ Key Laboratory of Systems Biomedicine (Ministry of Education) Shanghai Centre for Systems Biomedicine Shanghai Jiao Tong University Shanghai China; ^2^ Genome Analysis Laboratory of the Ministry of Agriculture Agricultural Genomics Institute at Shenzhen Chinese Academy of Agricultural Sciences Shenzhen China; ^3^ Department of Computer Science and Technology School of Electronic and Information Engineering Xi'an Jiao Tong University Xi'an China; ^4^ GenePlus‐Beijing Beijing China; ^5^ The Department of Obstetrics and Gynecology Xinhua Hospital affiliated to Shanghai Jiao Tong University Shanghai China; ^6^ The Department of Obstetrics and Gynecology Shenzhen People's Hospital Shenzhen China; ^7^ Shanghai FLY Medical Laboratory Shanghai China; ^8^ Comparative Pediatrics and Nutrition Department of Veterinary and Animal Sciences Faculty of Health and Medical Sciences University of Copenhagen Frederiksberg Denmark

**Keywords:** cervical cancer, cervical intraepithelial neoplasia, DhMR, DMR, hydroxymethylation, methylation

## Abstract

**Background:**

Cervical cancer (CC) is the second leading cause of cancer death among women worldwide. Epigenetic regulation of gene expression through DNA methylation and hydroxymethylation plays a pivotal role during tumorigenesis. In this study, to analyze the epigenomic landscape and identify potential biomarkers for CCs, we selected a series of samples from normal to cervical intra‐epithelial neoplasia (CINs) to CCs and performed an integrative analysis of whole‐genome bisulfite sequencing (WGBS‐seq), oxidative WGBS, RNA‐seq, and external histone modifications profiling data.

**Results:**

In the development and progression of CC, there were genome‐wide hypo‐methylation and hypo‐hydroxymethylation, accompanied by local hyper‐methylation and hyper‐hydroxymethylation. Hydroxymethylation prefers to distribute in the CpG islands and CpG shores, as displayed a trend of gradual decline from health to CIN2, while a trend of increase from CIN3 to CC. The differentially methylated and hydroxymethylated region‐associated genes both enriched in Hippo and other cancer‐related signaling pathways that drive cervical carcinogenesis. Furthermore, we identified eight novel differentially methylated/hydroxymethylated‐associated genes (*DES, MAL, MTIF2, PIP5K1A, RPS6KA6, ANGEL2, MPP*, and *PAPSS2*) significantly correlated with the overall survival of CC. In addition, no any correlation was observed between methylation or hydroxymethylation levels and somatic copy number variations in CINs and CCs.

**Conclusion:**

Our current study systematically delineates the map of methylome and hydroxymethylome from CINs to CC, and some differentially methylated/hydroxymethylated‐associated genes can be used as the potential epigenetic biomarkers in CC prognosis.

## Graphical abstract

1. The differentially DMR/DhMR occurred earlier and much more than that of CNVs which would be more suitable for a marker to detect the disease progression.
2. The differentially DMR/DhMR‐associated genes enrich in Hippo and other cancer‐related signaling pathways.
3. Eight DMR/DhMR‐associated genes significantly correlate with the overall survival of CC.

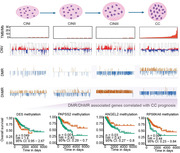

## 1 BACKGROUND

Cervical cancer (CC) is one of the most common gynecological tumors that become a health threat to women. There are more than half a million new cases and more than 300,000 deaths worldwide each year.^1^ China is one of the countries with a high incidence of CC due to the lack of a large‐scale standardized regular screening and the low usage of human papilloma virus (HPV) vaccine.^2^ Although, the 5‐year survival rate of CC patients detected at an early stage is more than 90%, the survival rate decreases dramatically for advanced CC and metastatic CC.^3^ Therefore, it is urgent need to further study the pathogenesis of CC and to find potential biomarkers to research and develop novel diagnostic techniques and treatments for advanced CC and metastatic CC.

It is reported that aberrant epigenetic modifications, including primarily cytosine methylation (5mC) and hydroxymethylation (5hmC) can lead to inappropriate activation/suppression of genes and then drive the tumorigenisis.^4^, ^5^ While DNA 5mC has been implicated in numerous biological processes, and aberrant DNA methylation patterns are considered as a hallmark of cancer, the biological significance of DNA 5hmC in cancer remains elusive. Recent studies have shown global loss of 5hmC in a variety of human solid tumors (breast, colon, gastric, liver, lung, melanoma, and prostate cancer) compared with the normal surrounding matched tissue demonstrated by immunohistological chemistry and dot blot assays.^6^, ^7^ The depletion of DNA 5hmC in cancer may also be a potential biomarker for early detection and prognosis of distinct cancers.

In CC, one of the fundamental processes driving the initiation and progression of CC is the accumulation of genetic and epigenetic alterations in cervix epithelial cells. A large amount of evidence showed that the methylation of the host tumor suppressor gene promoter region can cause dysregulation of many genes which in turn leads to cervical tumorigenesis.^8^ Verlaat et al reported that cancer‐like methylation patterns in CC could be detected early in cervical intra‐epithelial neoplasia (CIN) using 12 host‐cell DNA methylated genes.^9^ Furthermore, improvements in the methods used for detecting DNA methylation and hydroxymethylation have accelerated our understanding of epigenetic variants in cancers. Wang et al drew the whole 5mC and 5hmC profile in CC and compared to cervicitis tissues by semi‐quantitative methylation analysis.^10^ However, these studies did not draw a full epigenomic map that can differentiate 5mC from 5hmC in the entire pathogenesis from normal to CIN to CC.

In addition, multi‐omics integrated analysis can better illuminate the mechanism of the occurrence and development of carcinoma. Integrated analysis of methylation and gene expression for diagnosis, treatment, or prognosis has been reported in breast cancer, lung cancer, thyroid cancer, and hepatocellular carcinoma, head and neck squamous cell carcinoma, and polycystic ovary syndrome.^11^, ^12^, ^13^, ^14^, ^15^, ^16^ Although CC screening programs are now carried out worldwide, many women are still diagnosed with advanced CC which the overall prognosis remains poor. To date, only a limited number of reports about biomarkers were identified in CC by multi‐omics integrated analysis.

In this study, we systematically analyzed the landscape diagram of methylation and hydroxymethylation from normal to CIN to CC at single‐base resolution using whole‐genome bisulfite sequencing (WGBS‐seq) and oxidative WGBS (oxWGBS‐seq) methods. In addition, we also performed an integrative analysis of WGBS‐seq, oxWGBS‐seq, RNA‐seq data and histone modifications profiling data from TCGA to identify CC‐specific potential epigenomic biomarkers.

## 2 RESULTS

### 2.1 A decreasing trend of global 5mC and 5hmC levels from healthy groups to CCs

We performed WGBS‐seq and oxWGBS‐seq on 16 genomic DNA extracted from two health cervical (healthy) tissues, six CIN tissues (CIN1, CIN2, CIN3, *n* = 2 for each stage), and four paired CC tissues, and adjacent paracancer tissues (Figures S1 and S2). The adequate and enough DNA with good quality were sequenced to a depth of 10.48‐fold and 10.44‐fold for WGBS‐seq and oxWGBS‐seq with average cytosine coverage of 96.03% and 95.69%, respectively (Table S2). We observed both high bisulfite (unmethylated cytosine to uracil, 99.92%) and high oxidative bisulfite conversion rates (5‐hydroxymethylcytosine to uracil, 96.57%; Table S2). In total, 4.8–14.8 million CpG sites could be used to evaluate the hydroxymethylation variation at base‐resolution across all samples. Thus, we created genome‐wide, single‐base‐resolution 5mC and 5hmC maps of the healthy groups, CINs, and CCs, and we drew a map of 5mC and 5hmC modification profiling in CpG contexts (Figure S3).

**FIGURE 1:**
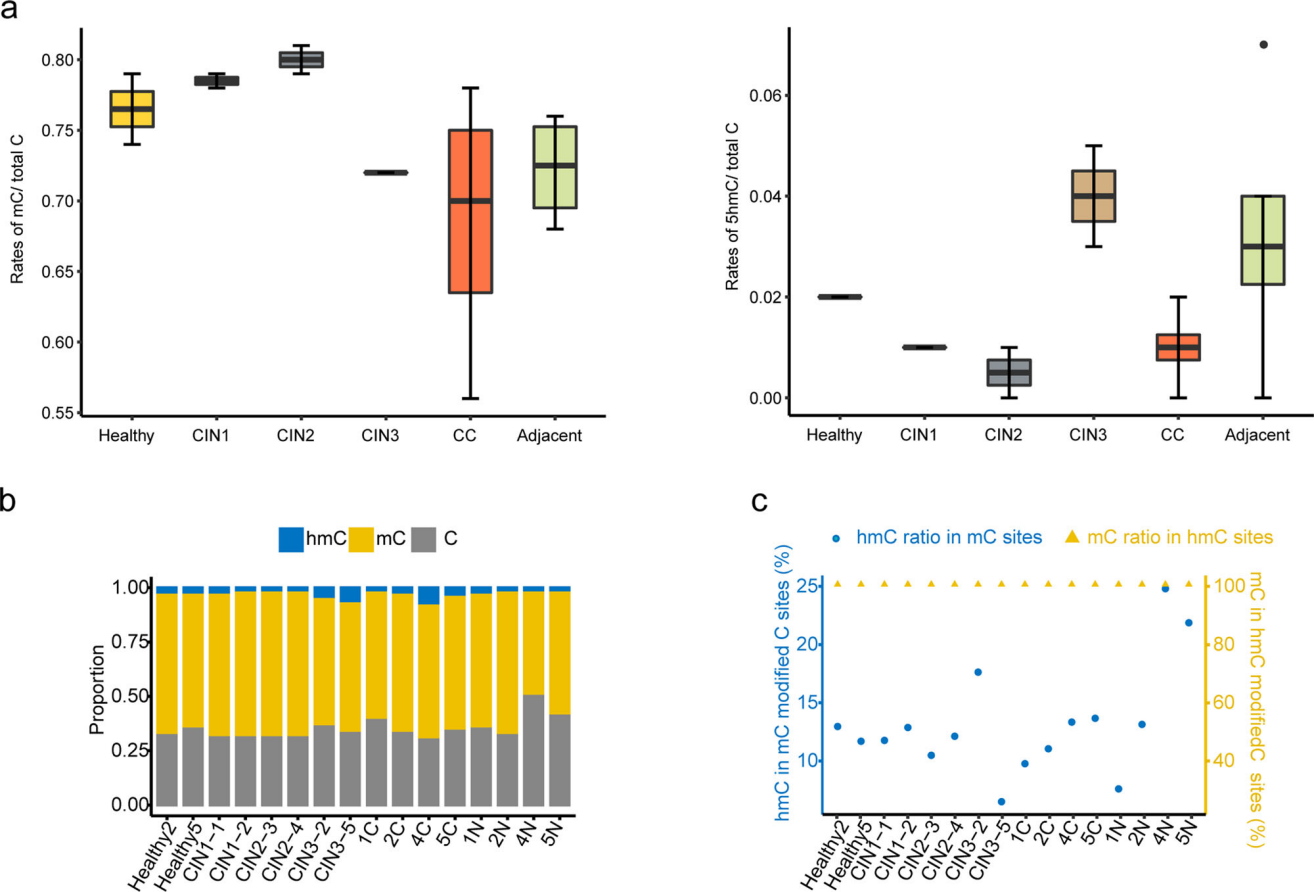
Global DNA methylation and hydroxymethylation profile in cervical cancer. (A) Average global methylation (left) and hydroxymethylation (right) levels of all CpG sites across the genome. (B) Absolute cytosine modification levels (C, mC, hmC) at individual CpG sites in each sample. (**C)** The ratio of 5hmC in 5mC modified sites and the ratio of 5mC in 5hmC modified sites. Abbreviations: CIN, cervical intraepithelial neoplasia; CC, cervical cancer; 5mC, 5‐methylation cytosine; 5hmC, 5‐hydroxymethylcytosine

We then compared the global levels of 5mC and 5hmC in healthy groups, CINs and CCs according to beta values derived from WGBS‐seq and oxWGBS‐seq libraries (see methods for details, the level was defined as the ratio of methylated or hydroxymethylated cytosines to the total number of cytosines). On the genomic scale, the average global 5mC levels at CpG sites were 73.67 (56.20%–80.97%), and the average global 5hmC levels at the CpG sites were 2.39% (0.75%–7.23%) among 16 samples. Both 5mC and 5hmC levels showed a decreasing trend from healthy groups (72.45% and 3.31%) to CCs (68.66% and 0.94%), even if the level of 5mC and 5hmC in the adjacent para‐cancer tissues tended to be higher than that in the cancer tissues (Figure 1A). The average 5mC levels were 76.58%, 78.57%, 79.84%, and 72.13% for healthy, CIN1, CIN2, and CIN3, respectively. While the average 5hmC levels were 1.83%, 1.141%, 0.82%, and 3.62% for healthy, CIN1, CIN2, and CIN3, respectively.

In addition, we also analyzed the distribution frequency of methylation, hydroxymethylation, and unmethylation at modified CpG site. The 5hmC level was 43‐fold lower than the 5mC level across all the patients (Figure 1B). We found that there were 6.33%‐24.63% of 5hmC modifications in all of 5mC modificated sits in all samples, while about 10% of 5hmC modifications in healthy cervix (Figure 1C, blue marked). Interestingly, we found that 100% 5hmC sites were also simultaneously modified by 5mC (Figure 1C, yellow marked). Our results provide valuable data in support of the reports that upstream modification of hydroxymethylation is methylation.^17^, ^18^

### 2.2 Distribution of 5mC and 5hmC content in human genomic regions

**FIGURE 2:**
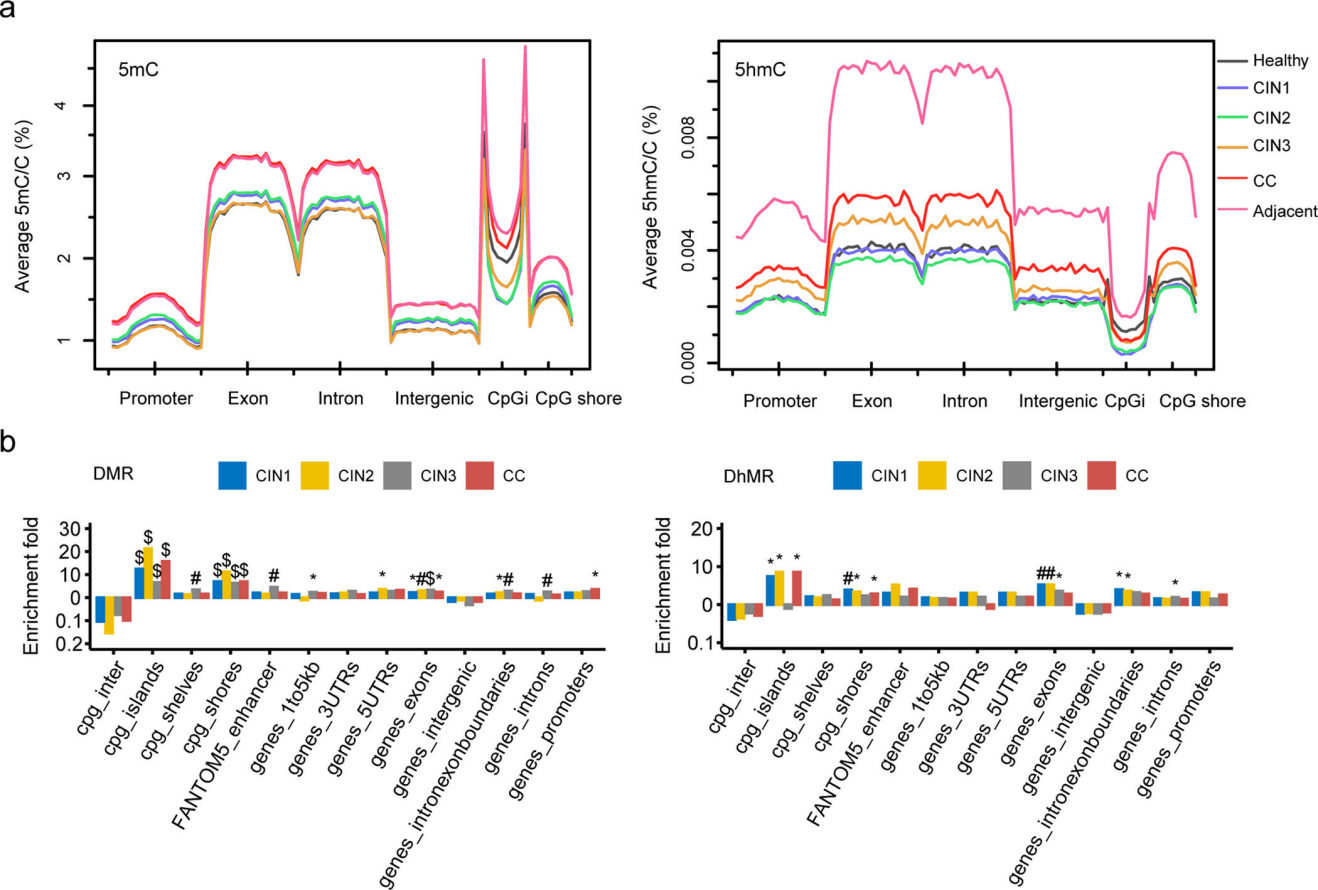
Genomic features relative density of 5mC (A, left) and 5hmC (A, right) within function genomic regions in hg19. CpGi, CpG island. The enrichment fold in each functional region was determined by the ratio of random simulated regions to the DMR (B, left) or DhMR (B, right) within the regions and the DMR/DhMR without the regions. *p* values were calculated by hypergeometric's test. Cpg_inter, cpg intergenic region. (*p < 0.05, #*p* < 0.01, $*p* < 0.001)

We then analyzed the distribution of 5mC and 5hmC in genomic regions in each group. Our results showed that 5mC sites and 5hmC sites distributed across the human genome (Figure 2A). To explore whether the methylation levels in a strand‐dependent manner, the methylation levels were interrogated by both Watson and Crick strand. The methylation level in both strands was plotted for each sample, and we did not observe any differences of methylation between the Watson and Crick strands for each sample (typical result was shown in Figure S4A). Intriguingly, from the kernel density of mC/hmC levels results, the ratio of fully‐methylated sites in the CC group was 47% lower than groups in the healthy and CINs. However, no differences were observed on the ratio of fully‐hydroxymethylated sites among the healthy, CINs, and CC stages (Figure S4B).

Consistent with the global methylation levels, the 5mC and 5hmC levels exhibited a similar trend in different genomic regions and all chromosome in CINs and CC. However, we observed different methylation patterns at locally functional regions including the exon and intron regions, promoter, and intergenic regions (Figure 2A).

### 2.3 The DMRs and DhMRs for cervical carcinogenesis

We identified a total of 201, 48, and 1,942 DMRs (differentially methylated regions) in CIN1, CIN2, and CIN3, while 1,290, 1,156, and 7,853 DhMRs (Differentially Hydroxymethylated Regions) in CIN1, CIN2, and CIN3 compared with healthy group, respectively. Four thousand five hundred eighty‐nine DMRs and 783 DhMRs were identified in CCs and adjacent paracancer tissues (Tables S3 and S4).

The DMR and DhMR were preferably enriched in CpG island (CpGi) and CpG shore (flanking ± 1 kbp of CpG island) (hypergeometric test, *p* < 0.05; Figure 2B). Remarkably, DMR of CINs and CC significantly enriched in exons (hypergeometric test, *p* < 0.05; Figure 2B), compared to the expected. DMR in CIN3 co‐localized in enhancers, flanking 1–5 kb of genes, exon‐intron boundaries and introns, while methylation in gene promoter was only significantly found in CC (hypergeometric test, *p* < 0.05; Figure 2B), suggesting the essential role of promoter methylation in CC initiation.

**FIGURE 3:**
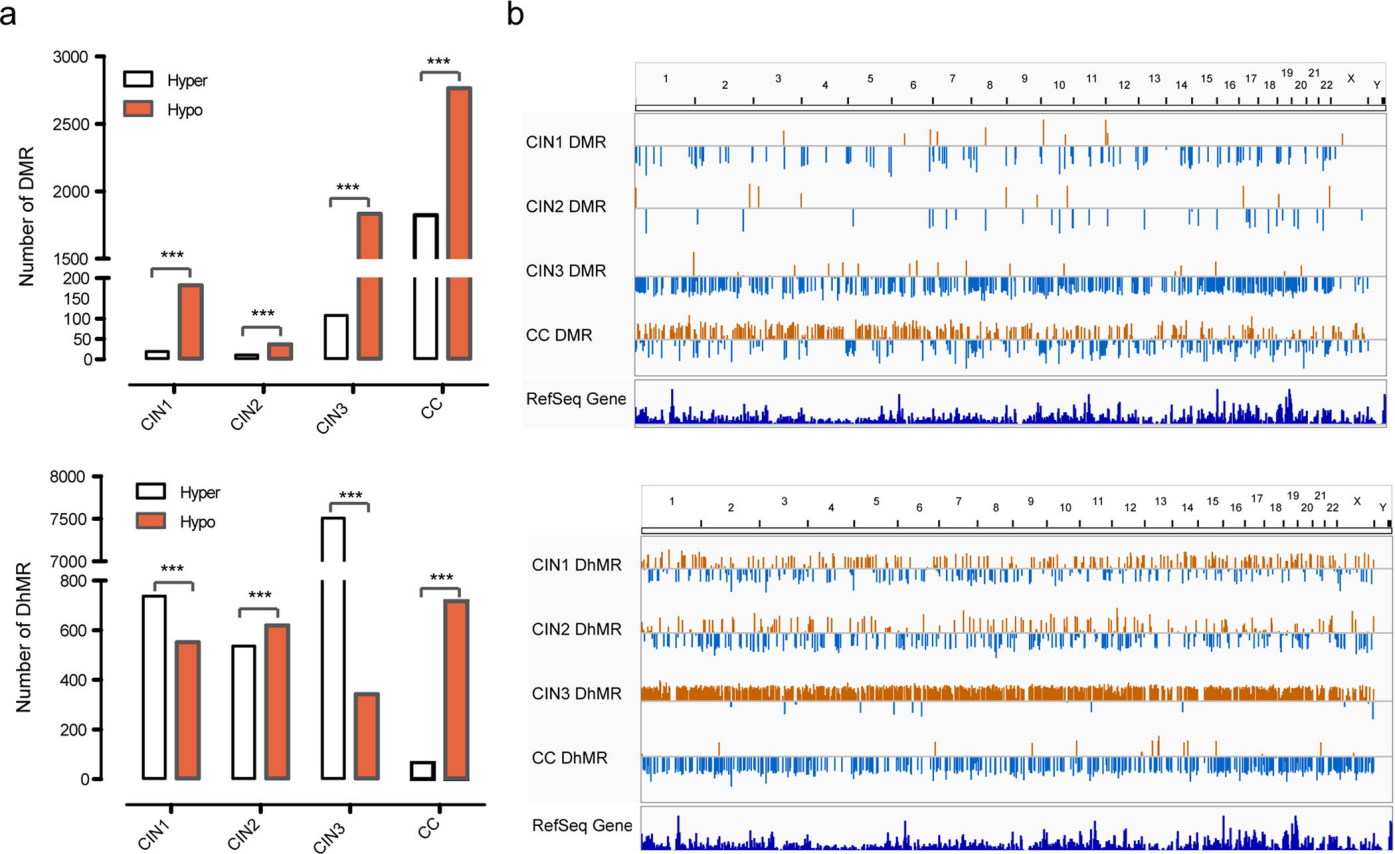
Genome‐wide DMRs and DhMRs in CINs and CC. (A) Number of regions of DMRs (up) and DhMRs (down) occur in CINs and CC. Regions that gain a mark (“hyper‐”) are represented by white bars, whereas losses (“hypo‐”) are marked by red. (B) Chromosomal distribution of significant DMRs (up) and DhMRs (down) compared with the healthy. The length of the line represents the level of methylation difference. Upward orange lines are hypermethylated regions, and downward blue lines are hypomethylated regions. (*p < 0.05, **p < 0.01, and ****p < 0.0001)

The number of DMR increased in CIN3 and CC stage, compared with CIN1 and CIN2, suggesting that the increased methylation changes in CC development. The number of DhMR increased dramatically in the CIN3 stage and decreased in CC stage (Figure 3A). Next, we also compared the distribution of DMRs and DhMRs among CC at the chromosomal level. The results suggested that CIN3 was mainly hypomethylated, while CC was mainly hypermethylated. Consistent with these results, high hydroxymethylation occurred in CIN3, and a low level of hydroxymethylation occurred in CC (Figure 3B). It is suggested that CIN3 displayed the most drastic demethylation in the process of CC.

### 2.4 Functionally associated genes based on DMRs and DhMRs

**FIGURE 4:**
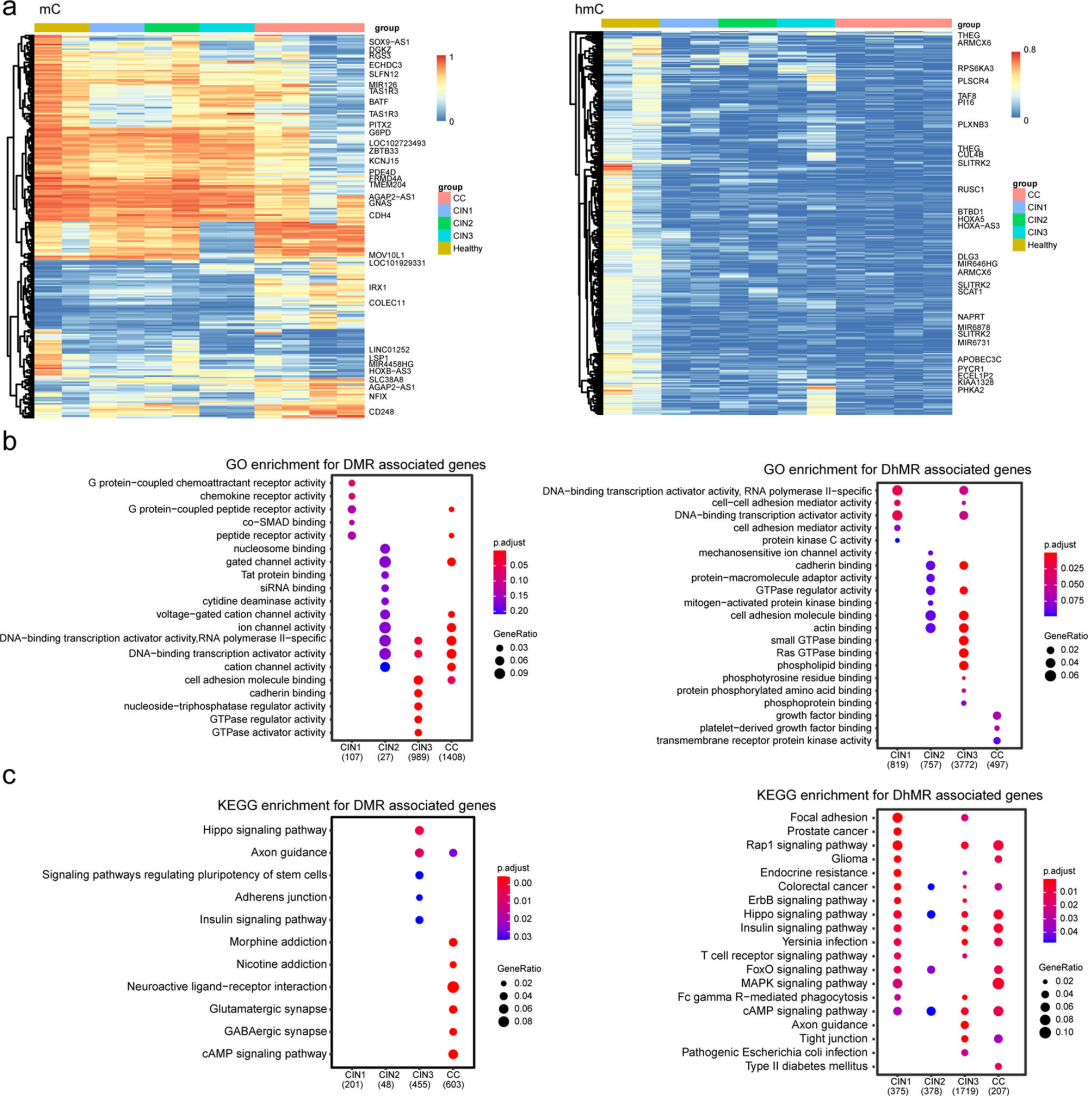
Cluster analysis of DMR and DhMR, GO and KEGG pathway analysis for DAGs based on DMRs and DhMRs. (A) Hierarchical clustering of DMRs (left) and DhMRs (right) methylation level in different groups during cervical carcinogenesis. Each row represents one DMR or DhMR. Hypermethylated DMRs are shown in red, hypomethylated DMRs in blue. The gene on the right indicates that its DMR or DhMR is located in the promoter region. (B) GO analysis for DAGs/DhAGs based on DMRs (left) and DhMRs (right). (C) KEGG pathway analysis for DAGs/DhAGs based on DMRs (left) and DhMRs (right). Groups are shown at the bottom. The total number of selected genes within the GO pathways on the dot plot is shown in brackets. The color of the dot indicates the adjusted *p*‐value (*p* < 0.05 and FDR < 0.05), and the size of the dot is proportional to the number of DEGs in the given pathway

We then annotated the genes associated with DMR and DhMR. Here, we called them DMRs‐associated genes (DAGs) or DhMRs‐associated genes (DhAGs). Considering the promoter methylation mainly contributes to the gene expression, we focused on the promoter‐linked DAGs and DhAGs, we found several genes (such as *NFIX, CDH4, PDE4D, PITX2*, and *G6PD*) were closely related to cervical carcinogenesis (Figure 4A left). These hypermethylation genes could play a role in the CC progression. Similarly, we also found two genes (*PLSCR4, CUL4B*) in hydroxymethylation (Figure 4A right). Of these genes, *NFIX* and *PITX2* might be used as early detection and prognostic markers for breast cancer.^19^, ^20^ *CDH4* and *PDE4D* can be used as early diagnostic markers of gastrointestinal tumor and prostate cancer, respectively.^21^, ^22^ Together, these findings provided further support for an important role of epigenesis in CC tumorigenesis and progression.

To gain insight into the potential biological function of methylation and hydroxymethylation, the DAGs and DhAGs were enriched via GO and KEGG. GO enrichment results showed that CINs and CC‐related biological processes including DNA−binding transcription activator activity and cell adhesion molecule binding (Figure 4B). The KEGG enrichment result showed that these potentially methylated genes were significantly enriched in Hippo, cAMP, Adherens junction, Axon guidance, and Neuroactive ligand‐receptor interaction (all *p* < 0.05); hydroxymethylated genes were also significantly clustered in Hippo, cAMP, Rap1, ErbB, and MAPK signaling pathways (Figure 4C) (all *p* < 0.05).

### 2.5 The relationship between methylation and hydroxymethylation level and gene expression in DAGs and DhAGs

**FIGURE 5:**
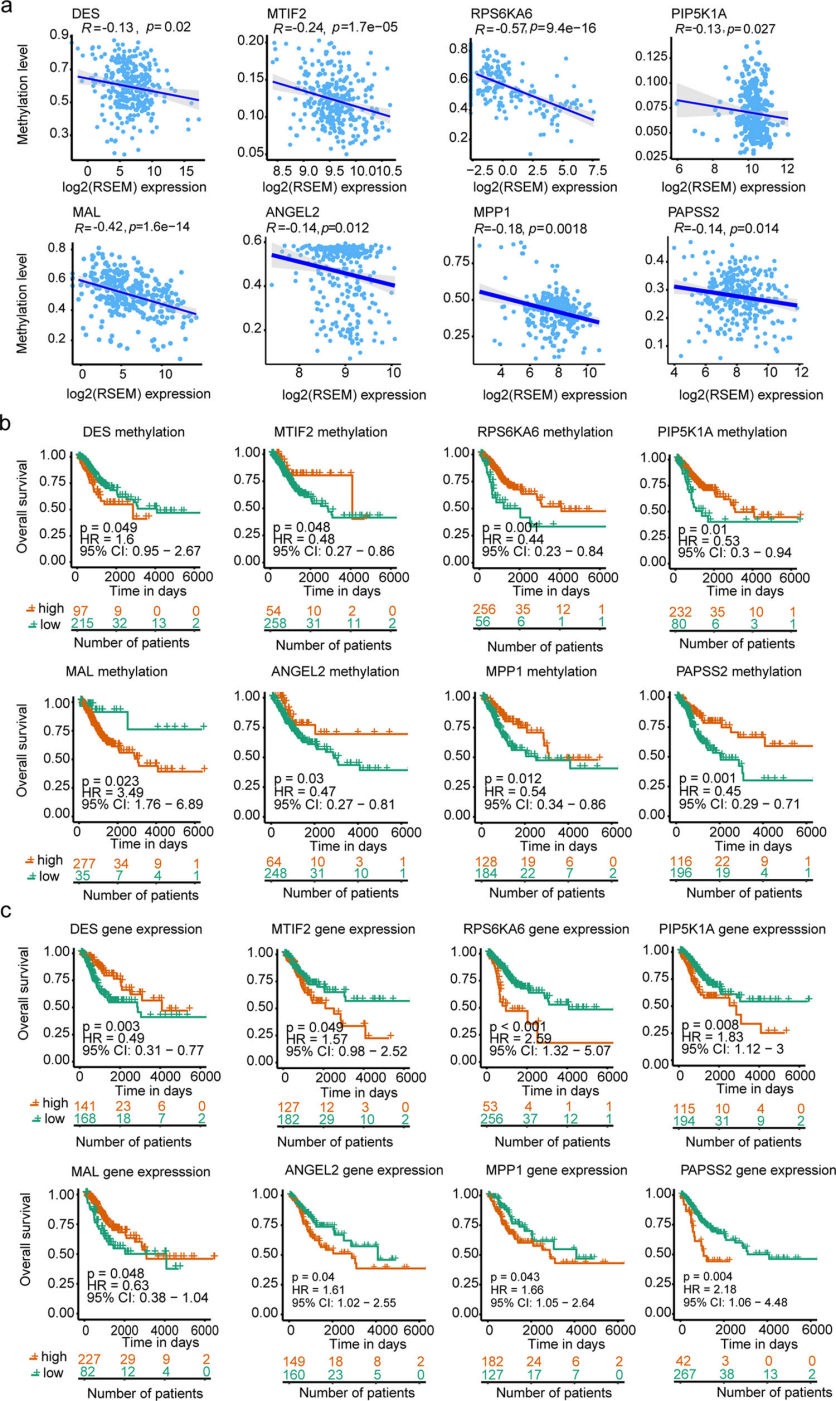
Correlation between DNA methylation and survival outcomes. (A) Spearman correlation was used to measure linear relationships between DNA methylation and gene expression levels. Statistically significant expression difference (log‐rank *p*‐value < 0.05) were found in eight genes. x‐axis: gene expression levels; y‐axis: DNA methylation levels. (B) Kaplan–Meier curves presenting the association of eight genes methylation and survival outcomes. Statistically significant survival difference (log‐rank *p*‐value < 0.05) were found in eight genes. x‐axis: Day; y‐axis: probability of overall survival. (C) Association of eight gene expression and survival outcomes. Red indicates the high methylation level or expression level; green indicates the low methylation level or expression level

To determine the association between the methylation/hydroxymethylation level and gene expression, we downloaded the level 3 methylation data and the paired RNAseq data of CC deposited in TCGA database. Then, we analyzed the expression level of DMRs/DhMRs‐associated genes (DAGs) from TCGA, of which these DAGs were overlapped to our results (Tables S3 and S4). And, we found that 67.61 % and 81.27 % for promoter and gene bodies of DMR/DhMR‐associated genes that identified in our data, were epigenetically silenced in TCGA through the correlation between methylation and expression.^23^ Typical results showed that gene expression was negatively correlated with methylation (Figure 5A). As an example, the *MAL* expression showed a significant negative correlation with its promoter methylation (Spearman *rho* = −0.42, *p*  =  1.6e−14). Besides, we also analyzed methylation and hydroxymethylation levels in promoters and exons in each stage of cervical lesions (Figure S5A). Higher levels of methylation were observed in upregulated gene expression at the promoter boundary. These observations were consistent with previous studies.^24^ Similarly, a positive trend was observed between methylation and gene expression in exon boundaries (±150 bp), especially in cancer and matched adjacent paracancer tissues. However, for hydroxymethylation, similar phenomenon was not found in any of these groups (Figure S5B).

To further understand the clinical relevance of DAGs/DhAGs in CC, the same strategy was performed as the above. We firstly identified DMRs/DhMRs‐associated genes (DAGs) from TCGA, of which these DAGs were overlapped to our results (Tables S3 and S4). And, then we investigated the association between the methylation or expression of these DAGs/DhAGs and overall survival. Both methylation and expression showed statistically positive correlation with the overall survival rate of *MTIF2*, *PIP5K1A*, and *RPS6KA6* in CC, whereas those of *DES* and *MAL* exhibited negative association with overall survival (all log‐rank *p* < 0.05 Figures 5B and 5C). Besides, we used the same analysis method for DAGs/DhAGs of CIN3. Interestingly, three genes (*ANGEL2, MPP1*, and *PAPSS2*) were also identified with statistically significant differences in the overall survival for CC. Therefore, methylation of these eight genes could be used as potential biomarkers for predicting prognosis.

### 2.6 Integrated analysis of methylation/hydroxymethylation and genomic variations

**FIGURE 6:**
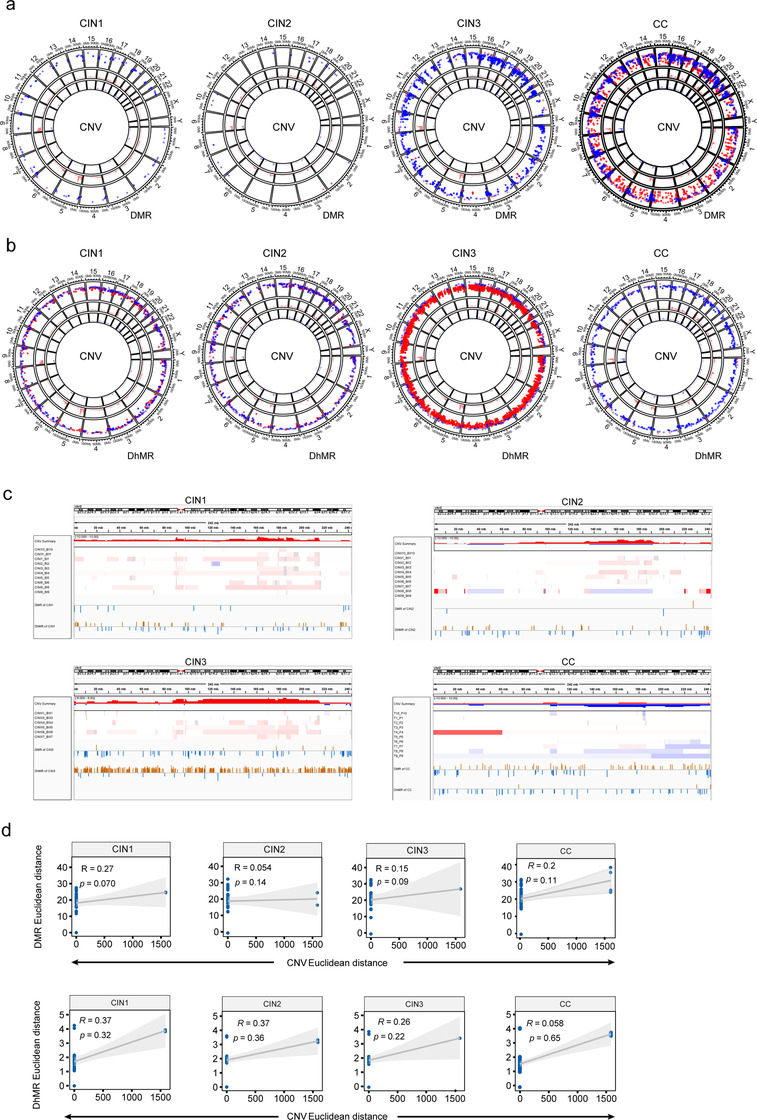
Integrated analysis of methylation/hydroxymethylation and genomic variations. (A) Circos diagram depicting distribution of between methylation changes and CNV alterations across the genome in CIN1, CIN2, CIN3, and CC group. Circular tracks from inside to outside: CNV, CNV, methylation changes, genome positions by chromosomes; red, gain; blue, loss. (B) Circos diagram describing the distribution of between hydroxymethylation changes and CNV alterations in CIN1, CIN2, CIN3, and CC group across whole genome. (C) IGV shows the distribution of CNV and methylation/hydroxymethylation in chromosome 2. (D) The correlation of CNV (z score of log2 read density) and methylation or hydroxymethylation (z score of M values; lower panel) in CIN1, CIN2, CIN3, and CC group

To investigate the relationship among methylation/hydroxymethylation levels and copy number variations, we selected CNV data using both the bisulfite sequencing data and the whole exome sequencing of CC and CINs in our previous study.^25^ We compared the locations of CNV regions and methylation regions unmatched data. The SCNV gain or loss region did not consistently overlap with DMR regions across the genome (Figures 6A and 6B). And an example showed the inconsistency of the location of CNV and DMR/DhMR in chromosome 2 (Figure 6C).

To explore the association between CNV and methylation/hydroxymethylation, the genome was randomly divided into constant bins (size 33 kb, optimized by ReadDepth), and the read density and methylation/hydroxymethylation was measured using bisulfite sequencing data. We used M values (log2 of beta values) to represent methylation/hydroxymethylation levels in the bin size. Using the z score of log2 (copy number ratio) and methylation/hydroxymethylation variation,^26^ the correlation of CNV and methylation/hydroxymethylation was measured for all samples. A negligible or weak correlation was found between CNV and methylation/hydroxymethylation (Spearman correlation coefficiency, range −0.0078 ∼ 0.36, mean 0.17 and −0.073 for CNVvsmC and CNVvshmC) in all the matched data. And, the correlation coefficiency and *p* values were inconsistent across samples at specific stage (Figure S6A).

To further assess the relationship between CNV and methylation, we measured the euclidean distance between pairwise CNV and methylation at different stages using bisulfite sequencing data. And, the correlation coefficiency were 0.27 (*p* = 0.070), 0.054 (*p* = 0.14), 0.15 (*p* = 0.09), 0.20 (*p* = 0.11) between the CNV and methylation distance for CIN1, CIN2, CIN3, and CC, respectively (Figure 6D). The correlation coefficiency was 0.37 (*p* = 0.32), 0.37 (*p* = 0.36), 0.26 (*p* = 0.22), and 0.058 (*p* = 0.65) between CNV and hydroxymethylation for CIN1, CIN2, CIN3, and CC, respectively (Figure 6D). Taken together, no significant correlations were observed between CNV and methylation/hydroxymethylation in the CINs and CC.

In terms of the degree of change, the methylation/hydroxymethylation occurred earlier and much more than that of CNV (Figures 6A and 6B, and S6B), which would be more suitable for a marker for early warning of CC. In addition, the mutation types in cytosine sites were measured according to methylated status using WGBSseq and oxWGBSseq data. Hydroxymethylated cytosines were highly enriched in C > A and C > G transversions (all *p* < 0.001), while C > A mutations were also observed at methylated cytosines (*p* < 0.01; Figure S7).

### 2.7 Integrated analysis of methylation and hydroxymethylation with histone modifications peaks

Finally, we associated the methylation and hydroxymethylation with histone modification. The methylation and hydroxymethylation levels were mapped to signals of ChIP‐Seq dataset of six epigenetic marks in the normal human cervix from the ENCODE depository.

**FIGURE 7:**
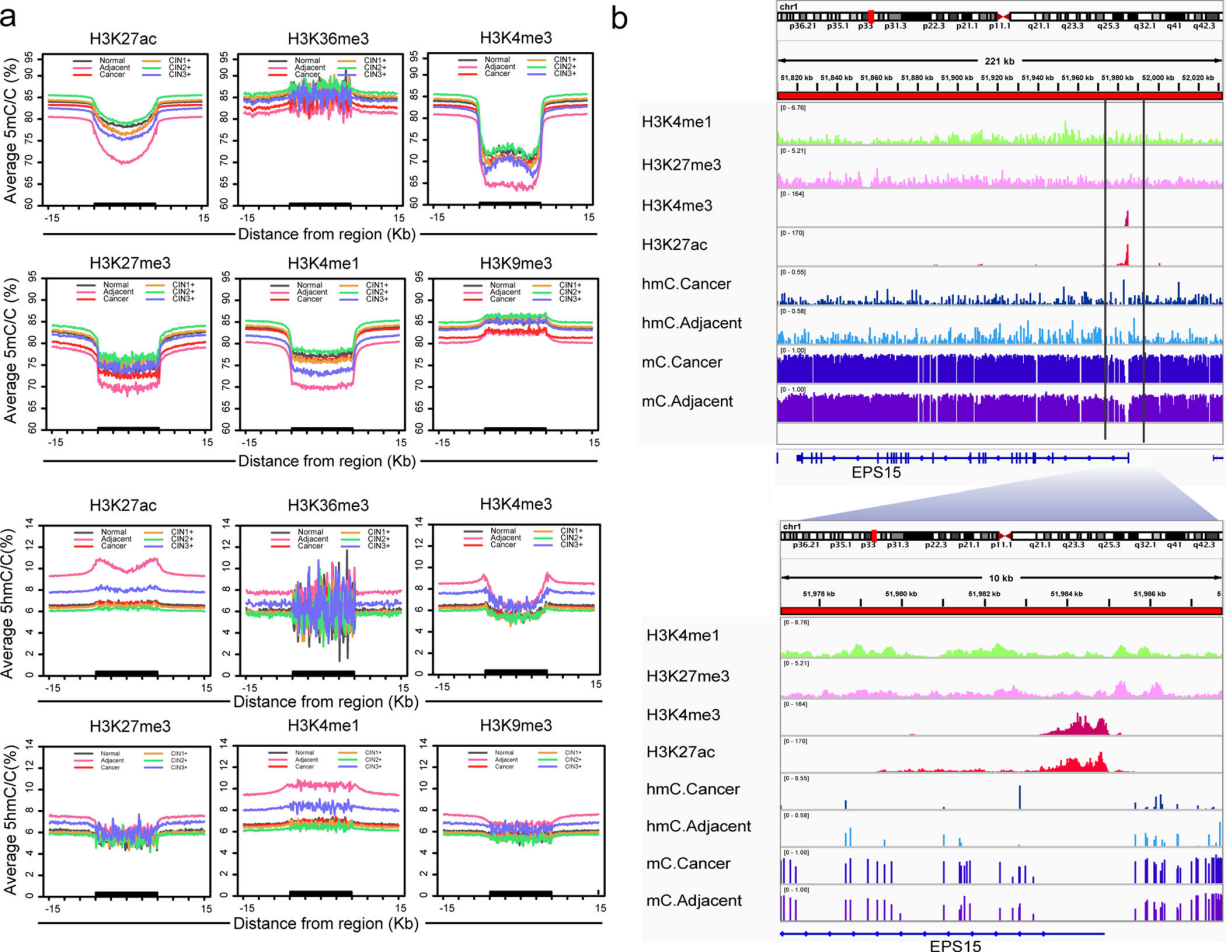
Integrated analysis of methylation/hydroxymethylation and histone. (A) 5mC (up) and 5hmC (down) density distribution across histone modifications and their flanking regions in healthy, CINs, and CC. The x‐axis represents the histone peaks and their 15 kb flanking region. Black bold on the x‐axis indicates histone peaks/domains. The y‐axis represents the methylation level in the corresponding region. (B) Profiles of 5mC, 5hmC, and major histone modification occupancy for EPS15. An example of a 200‐kb genomic region surrounding the EPS15 gene showing enrichment of H3K4me3 and H3K27ac within the genic region and enrichment of 5mC and 5hmC in the neighboring intergenic regions

Promoter‐ and enhancer‐ associated histone binding peaks (peaks ± 5 kb, ranging ±15 kb) were associated with DNA methylation. H3K4me3 was depleted both in DNA methylation and hydroxymethylation. H3K27ac and H3K4me1 were depleted in methylation, while enriched in hydroxymethylation (Figure 7A). Especially, methylation levels in promoter‐associated H3K4me3 and enhancer‐associated marks (H3K27ac and H3K4me1) were significantly lower in CIN3 and adjacent paracancer tissues than in tissues of healthy and CINs. H3K27me3, a poly‐comb‐associated mark,^27^ was only depleted in methylation. No discrepancy was observed in methylation and hydroxymethylation at H3K36me3 peaks, which is a dsDNA repair‐marks,^28^ among all cervix tissues. The association of DNA methylation and hydroxymethylation as well as four‐related histone modification localized at enhancer region, the *EPS15* gene was shown as an example in both CC and paracancer tissues (Figure 7B). Our analysis suggested that methylation and hydroxymethylation were an epigenetic mark in cervical tissues. And the hydroxymethylation resisted methylation in DNA modification at epigenetic binding sites. An enrichment of hydroxymethylation in promoter and enhancer elements indicated its gene regulatory activity.^29^

## 3 DISCUSSION

Epigenetic modification, especially DNA methylation and DNA hydroxymethylation, as an epigenetic hallmark has been proved to play an essential role in cancer. Thus far, studies have reported that whole‐genome methylation and hydroxymethylation profiling for several cancers, profiling for CC remains limited. In this study, we have performed a base‐level analysis of DNA methylation and hydroxymethylation across the whole genome in healthy, CINs, and CC with matched adjacent paracancer tissues, which revealed novel insights regarding the role of epigenetic modification contributes to the cervical carcinogenesis and its prognosis.

The global methylation level of tumor tissues and the matched adjacent paracancer tissues are the same, but tend toward be higher than CINs and healthy; the global hydroxymethylation level of adjacent paracancer tissues tends toward to be higher than tumor tissues (Figure 2A). The results showed that although the morphology of the adjacent paracancer tissues had not changed, the epigenetics has changed on a large scale. And these observations are consistent with most of the previous work about cancer epigenetics.^30^, ^31^ Global hypo‐methylation and hypo‐hydroxymethylation have been observed in CC, especially, the number of DMRs and DhMRs increased in CC, compared with the healthy. The alteration of methylation and hydroxymethylation occurred in CpG islands and CpG shores in CINs and CC tissues. And the exon methylation was observed in CINs and CCs (Figure 2B). There results indicated that the local methylation/hydroxymethylation changes might greatly contribute to cervical carcinogenesis.

Locally hyper‐methylation and hyper‐hydroxymethylation have been identified in the CINs and CC. When CC compared to normal tissue, high hydroxymethylation occurred in CIN3 and low hydroxymethylation occurred in CC which suggested drastic transformation have taken place in the process of CC development (Figure 3A). A large number of hyper‐DMRs and hypo‐DhMRs occurred in CC, and amount of hypo‐DMRs and hypo‐DhMRs occurred in CIN3 (Figure 3B). The change of DMR and DhMR from CINs to CC exhibited a non‐linear pattern, rather than linear fashion.

Methylation and hydroxymethylation in lesions at CINs may be a key checkpoint for the fate of cervical cells. We found that methylation modification levels in stage CIN3 were distinct from CIN1‐2 stages with aberrant decreased methylation and sharply increased hydroxymethylation. Additionally, the differential methylation regions in CIN3 specifically co‐localized in enhancers. It used to be thought that methylation/hydroxymethylation level of CIN progressed through these pre‐cancerous stages toward cancer in a linear fashion,^32^ except report of the disease progression in CC.^33^ Coincidently, the researchers use this model on the methylation status of the healthy individual, CIN1, CIN2+ and CC patients, and observed the non‐linear and bi‐modal methylation dynamic with maximum methylation in CIN2+ stage. Our study provides a more precise transition stage in the methylation dynamic process from CINs to CCs. Moreover, our results showed that a higher deviation of hydroxymethylation was observed than that of methylation in this process. Inspired by the previous studies,^34^ we suspected that CIN3 is a critical stage that the methylation/hydroxymethylation status is reversible in this stage and could determine the clinical outcome of CC. Moreover, the hydroxymethylation level might be more sensitive than methylation in this process due to its high deviation.

Several DMR‐related genes have been used as diagnostic or prognostic biomarkers in CC. Previous studies have reported several CC‐associated methylation genes including *SEPTIN9*, *TAFA4* (also known as *FAM19A4*), *CDKN2A* (coding p16^INK4a^), *SOX17*, *EZR* (coding Ezrin), *PAX1*, *SOX11*, *TERT*, *SOX1*, *LMX1A*, *FHIT*, *POU4F3*, *ADCY8*, *CDH8*, *ZNF582*, *ESR1*, *MYOD1*, *COL17A1*, *JAM3*, *ASCL1*, *LHX8*, and *ST6GALNAC5*.^35^, ^36^, ^37^, ^38^, ^39^, ^40^, ^41^, ^42^, ^43^, ^44^, ^45^, ^46^, ^47^, ^48^, ^49^, ^50^ Of these methylated‐genes related to CC, all of these reported genes were identified in our results (Tables S3 and S4). *ZNF582* and *PAX1* promoter methylation detection have been commercially used for CC diagnosis.^51^ The methylation of p16^INK4a^, which has been proved to be indirectly caused by HPV E7 oncoprotein,^52^ was found in CC. Besides, long non‐coding *AFAP1‐AS1* and *MEG3* ^53^, ^54^ that predicted the CC survival was also validated in our cohort (Tables S3 and S4). However, *CADM1*, which has been found to be methylation in CINs and CC were only found in CIN1 of our cohort.^55^ Intriguingly, *BRMS1* ^56^ and *RAD51B* ^57^ that were reported to be methylated as detected by qMSP or methylation microarray in CC were actually hyper‐hydroxymethylated in cancer and CIN3 of our cohort.

Notably, novel methylated genes were identified in CC. Typically, cancer driver genes *NFIX* and *CDH4* may act as methylation biomarkers for early detection for breast cancer and gastrointestinal tumorigenesis.^19^, ^21^ *PITX2* acts as an oncogene in multi‐cancers, while no reports about the role of methylation in other cancers as well as CC. As a whole, these novel methylated genes provided new insights for a better understanding of the progress of CC and may serve as useful biomarkers for the accurate management of CC.

GO and KEGG pathway analysis shows that the DAGs/DhAGs were significantly enriched in Hippo signaling pathways and human papillomavirus infection pathways. Hippo hijacks EGFR and HPV E6 oncoprotein to promote CC,^58^ and its downstream YAP and TAZ proteins play a role in cervical tumor‐infiltrated cell activation.^59^ Previously study finds that mutation, amplification, and deletions of Hippo in hypermethylated CC subgroup.^60^ We provided additional evidences that methylated genes in Hippo pathway occurred in CC, even at CINs stages. In addition, this finding is not similar to previous studies which have suggested that the cell cycle was a key biological process and a critical driver in CC by bioinformatics analysis.^61^ To some extent, our finding shows the advantage of finding the CC signal pathway by WGBS‐seq and oxWGBS‐seq.

Gene expression was controlled by multiple aspects including DNA methylation, DNA hydroxymethylation, histone modifications, thus, the correlation between DNA methylation, and gene expression might be overestimated when all genes were involved in the correlation analysis. It can thus be suggested that the alteration in gene expression and changes in methylation might be associated with the outcome of CC. Using TCGA as an external validation, we identify the epigenetic changes that may play a key role in the prognosis of CC. We finally identified eight DMR/DhMR‐related genes (*DES, MAL*, *MTIF2*, *PIP5K1A*, *RPS6KA6*, *ANGEL2, MPP*, and *PAPSS2*) that showed significant associations between overall survival and the identified DNA differential methylation‐related genes among the CC patients from TCGA. All the eight DMR/DhMR‐related genes were novel prognostic genes at methylation level in CC. As a tumor suppressor gene, the promoter methylation of *MAL* has been found in CINs and CCs.^55^ Hypermethylation of *MAL* is correlated with its downregulation of gene expression in esophageal adenocarcinomas.^62^ *PIP5K1α* and *PIP5K1A* have been reported to be involved in carcinogenesis.^63^, ^64^ *RPS6KA6* (*RSK4*) is a putative tumor suppressor gene.^65^, ^66^ The *RSK4* gene has also been reported as oncogenic gene in several cancers.^67^ Overexpression of *RSK4* positively correlates with poor prognosis in RCC and ESCC.^67^, ^68^ Notably, the roles of *DES* and *MTIF2* at genetic or epigenetic levels are unclear in human cancers.

The three prognostic markers (*PAPSS2, ANGEL2*, and *MPP1*) that hyper‐hydroxymethylated in CIN3 also play important roles in cancers. *PAPSS2* is critical for breast cancer cell migration and metastasis.^69^ Abnormal *ANGEL2* expression will affect the efficiency of pre‐tRNA processing.^70^ *MPP1* may be a new protein interaction target for therapy against tumors.^71^ The epigenetic modification of these genes needs to be further investigation in cancer biology and clinical implication.

The genetic and epigenetic variations co‐contribute to cervical carcinogenesis. Herein, we found that C > A and C > G mutations in hydroxymethylated sites were higher than non‐hydroxymethylated cytosines (Figure S7). These observations demonstrated an association between DNA mutation and modification, and these associations was also reported in human genome of cell lines, brain, kidney, and myeloid cells.^72^, ^73^ However, we found that methylation/hydroxymethylation and CNV were independent variables in our cohort, in line with previous results that methylation levels unrelated with CNV changes.^74^ In addition, we predicted the performance of methylation and hydroxymethylation are better than that of CNV in the diagnosis for cervical carcinogenesis. This could be due to epigenetic alterations that have been indicated to occur much earlier than genetic alterations in CC.^75^

## 4 CONCLUSION

In this study, we performed the genome‐wide profiles of DNA methylation and hydroxymethylation at single‐base resolution in the healthy, CINs lesions, CC. We found several genes that provide further support for the importance of epigenetics in tumorigenesis and progress. Through multi‐omics analysis, 5mC, and 5hmC might be irrelevant to CNV in cervical carcinogenesis. Finally, we identified eight novel prognosis‐associated genes and may serve as novel targets for CC treatment.

## 5 METHODS

### 5.1 Sample collection

For physical examination volunteers and patients suffering from CINs and CCs, the written informed consent was obtained from each person before enrollment. The cervical exfoliated cells were sampled using cervical brushes for each enrolled patient, which were used to observe the morphological characteristics based on the liquid‐based cytology (Becton Dickinson Company, New Jersey, USA). In Shenzhen People's Hospital, cervical liquid‐based cytological test was used as a routine screening test for CC. Hence, those patients with cytological abnormality were recommended to accept colposcopy examination. And then the colposcopy findings were used to determine if a biopsy is necessary. The diagnoses of CIN1, CIN2, CIN3, or CC were determined and reviewed by two pathologists independently.^76^ This study was approved by the Ethical Review Board of Shenzhen People's Hospital (SPH‐2017022).

A total of 16 samples including two healthy cervix tissues and six CINs and four CCs were enrolled in this study. The healthy cervix tissues and the paired adjacent paracancer tissues to CCs were used as the control. The biopsies from CINs and the surgically resected tumors and paired adjacent paracancer tissues were snap‐frozen in liquid nitrogen and stored at −80°C. All tissues were diagnosed by two independent pathologists using H&E staining. The purity of samples was above or equal to 70% according to pathological results, while no tumor content was observed in adjacent tumor tissues, CINs, and healthy cervix. All the CINs and cancer patients were HPV positive, while the healthy donors were HPV negative. A detailed description of clinical information was found in Table S1.

### 5.2 Methylome and hydroxymethylome sequencing

#### 5.2.1 WGBS‐seq and oxWGBS‐seq library preparation

The WGBS and oxWGBS libraries were prepared followed by the instruction of TrueMethyl Seq Kit (TrueMethyl Seq Kit, CEGX, Cambridge Epigenetix Limited). One microgram genomic DNA and non‐methylated lambda DNA were fragmented to 250 bp, then end‐repair step was performed in the presence of phosphokinase and DNA polymerase. The control DNA was spiked in to assess oxidative conversion rate. Then, repaired DNA was adding tailing A and adaptorization. The adaptorized libraries were purified with magnetic beads, denatured, and oxidized with or without KRuO4 to generate oxBS and BS libraries. After oxidation, the DNA was converted by sodium bisulfite and amplified with methylated primers. Then the digestion control was amplified and qualified with TaqαI enzymes. Finally, the libraries were amplified, qualified with bioanalyzer, and sequenced by Illumina Xten with PE150.

### 5.3 Methylome and hydroxymethylome bioinformatical analysis

Raw bisulfite sequencing data were filtered by cutadapt and trimgalore. Then clean data were mapped to reference hg19 using BSMAP software.^77^ The methylation levels at a single cytosine site represented as the beta value (the ratio of methylated cytosine count to total number of cytosine counts). The hydroxymethylation levels were obtained by mlml algorithm.^78^ The DMR and DhMR were analyzed by metilene,^79^ using circular binary segmentation with the following parameters and other default parameters. DMR was identified as differential methylated region with methylation levels > 0.2 and CpG coverage ≥ 5,^80^, ^81^ 2D‐KS and Mann–Whitney *U* test *p* < 0.001, and corrected by Benjamini‐Hochberg method. DhMR was defined as differential hydroxymethylated region with levels > 0.2, CpG coverage ≥ 5, mininum CpG ≥ 5 per region, valley filter = 0.05, 2D‐KS and Mann‐Whitney *U* test *p* < 0.001, and corrected by Benjamini‐Hochberg method.^79^ The number of DMR/DhMR varied dependent on the parameter thresholds; the number and landscape of DhMR with different parameters of two algorithms were shown in Figure S8 and Table S5.

### 5.4 Transcriptome data and correlation analysis

The RNA‐seq data of paired CC samples derived from our previously reported work was used to obtain the results in Supplementary Figure S5, ^25^ And, the RNA‐seq data were classified into high and low expression using absolute fold change of 2 as cutoff, while genes with no changes were defined as "no" change group. We calculated the methylation levels of all the genes localized around TSS or exons in the healthy, CINs, CC, and matched paracancer tissues.

For RNAseq from TCGA (*n* = 307), the RSEM value for each DAGs/DhAGs weas selected and correlated with the average methylation levels within DMR/DhMR for each gene with promoter methylation using Spearman coefficiency method.

### 5.5 SNV analysis using bisulfite sequencing data

The SNVs from WGBSseq and oxWGBSseq were obtained by Bis‐SNP using bisulfite sequencing data.^82^ Briefly, all the possible genotypes for each SNP were modeled using dbSNP data. Then the bisulfite data for each cytosine were used to establish Bayesian model using prior bisulfite conversion rate and probabilities of methylation level. Finally, the likelihood probability of SNP frequency was measured at the same loci by Bayesian interfere. We chose the SNVs that were identified both in bisulfite libraries and oxidative bisulfite libraries in the same sample to ensure the reliability. The overlapped SNVs were further filtered by 1000G, Esp6500, dbSNP150, Gnomad_exome, and exac03 datasets. Then the SNVs sites were counted by transversion/transition type, classified according to methylation status at cytosine sites, and viewed by ggplot2 package.

### 5.6 CNV analysis and correlation analysis

Copy number variation was called by Aberration Detection in Tumour Exome algorithm using whole‐exome sequencing data.^83^ The cutoff of copy number gain and loss were defined as absolute threshold ≥ 0.2. The copy number alterations were viewed through copynumber R package.

Copy number variation of bisulfite sequencing data was called by ReadDepth for single samples.^84^ Calling was detected by circular binary segmentation using the default parameters of software. The optimized bin was used as 33 kb for each samples. CNV gain and loss threshold was set as copy number ≥ 2.68 and copy number ≤ 1.38, respectively. Using the z score of log2 (copy number ratio) and methylation variation as follows,^26^ the correlation of pair‐wise CNV and methylation was measured for all samples using bisulfite sequencing data.

$$\begin{array}{ccc} Z_{\mathrm{meth}}\mathrm{score} & = & \left(M_{\mathrm{case}}-M_{\mathrm{normal}}\right)/SD_{\mathrm{case}};Z_{\mathrm{CNV}}\mathrm{score}\\ & = & \left(\log R_{\mathrm{case}}-\log R_{\mathrm{normal}}\right)/SD_{\mathrm{case}}. \end{array}$$

Whereas, M represent log2 of beta values. SD_case_ means the standard deviation of case sample. And logR depicts the log2 of read density.

### 5.7 Geneset enrichment analysis

The DMR/DhMR‐associated genes were annotated by GenomicRanges and genomation package. GO and KEGG pathway enrichment for DAGs/DhAGs of each disease stage was performed by clusterProfiler.^85^

### 5.8 Enrichment analysis

To understand the enriched functional region that DMR/DhMR preferred to occur in the genome, we randomly generate the same size of DMR/DhMR region as the expected regions. Then we counted the number of DMR/DhMR with or without specific functional regions (intergenic, CpG island, CpG shore, CpG shelves, FANTOM5_enhancer, gene bodies, 3′UTR, 5′UTR, intron, exon, flanking 1–5 kb of genes, boundary, and promoters) using annotatr package. Then the numbers of observed DMR/DhMR was compared with that of expected regions using hypergeometric test. The enrichment fold was calculated by the ratio of each functional region to the randomly simulated region.

### 5.9 Statistical analysis

Methylation levels were measured as the ratio of methylated cytosine to total cytosines at a single site. For comparison, the functional regions were divided into 20 or 10 bins. And the average methylation levels at functional regions including promoter, exons, introns, CpGi, CpG shores (URL:https://genome.ucsc.edu/cgi‐bin/hgTables), and enhancers were measured using genomation and GenomicRanges packages (R v3.6.1).^86^ The functional regions were annotated by annotatr R package.^87^

The average methylation level for each gene in DMR/DhMR was calculated using TCGA level3 methylation data (*n* = 307). The methylation level or gene expression was classified into a high and low group according to the optimal best cutoff point estimated using the survminer R package. The correlation with overall survival and gene expression levels was measured using the cox proportional hazard model, and statistical calculated by weighted log‐rank test and shown as Kaplan–Meier curve using the survival package.

## COMPETING INTERESTS

The authors declare that they have no competing interests.

## AUTHOR CONTRIBUTIONS

Jian Huang conceived the project, and Jian Huang and Fei Gao designed the experiments. Yanfang Guan, Pansong Li, Yanfang Guan, and Jiayin Wang performed sequencing assay. Yingxin Han, Liyan Ji, Mengya Ma, Yanfang Guan, Yanfang Guan, Yinge Xue, Yinxin Zhang, and Jian Huang analyzed the sequencing data. Wanqiu Huang performed the pathology experiments. Jian Huang, Fei Gao, Liyan Ji, Xipeng Wang, and Xin Yi contributed reagents, materials and analysis tools. Wanqiu Huang, Hong Xie, Li Jiang, Yuliang Deng, and Boping Zhou contributed the samples. Jian Huang, Yingxin Han, Yanfang Guan, and Liyan Ji integrated, analyzed, and interpreted all data. Jian Huang contributed to the supervision of the work. Jian Huang, Yingxin Han, and Liyan Ji wrote the manuscript with the assistance and final approval of all authors.

## ETHICS APPROVAL AND CONSENT TO PARTICIPATE

This study was approved by the Research and Ethical Committee of Shenzhen People's Hospital (SPH‐2017022) and complied with all relevant ethical regulations. Written informed consent was provided by all patients. All experimental methods abided by the Helsinki Declaration.

## Supporting information

figureS1‐S8

tableS1

tableS2

tableS3

tableS4

tableS5

## Data Availability

TCGA_CESC HM450k level3 data and RNAseq data were downloaded from firehose depository (http://gdac.broadinstitute.org/runs/stddata__2016_01_28/data/CESC/20160128/). The ChIPseq data of six histones modifications including H3K4me3, H3K27ac, H3K4me1, H3K36me3, and H3K9me3 were obtained from ENCODE (https://www.encodeproject.org/). The raw and processed oxWGBS‐seq and WGBS‐seq datasets generated in this study have been submitted to the NCBI Sequence Read Archive (http://www.ncbi.nlm.nih.gov/bioproject/662817; SRA: PRJNA662817).

## References

[1] Yang W , Lee KW , Srivastava RM , et al. Immunogenic neoantigens derived from gene fusions stimulate T cell responses. Nat Med. 2019;25:767–775. 31011208 10.1038/s41591-019-0434-2 PMC6558662

[2] Chen W , Zheng R , Baade PD , et al. Cancer statistics in China, 2015. CA Cancer J Clin. 2016;66:115–132. 26808342 10.3322/caac.21338

[3] Bray F , Ferlay J , Soerjomataram I , et al. Global cancer statistics 2018: GLOBOCAN estimates of incidence and mortality worldwide for 36 cancers in 185 countries. CA Cancer J Clin. 2018;68:394–424. 30207593 10.3322/caac.21492

[4] You JS , Jones PA . Cancer genetics and epigenetics: two sides of the same coin?. Cancer Cell. 2012;22:9–20. 22789535 10.1016/j.ccr.2012.06.008 PMC3396881

[5] Shen H , Laird PW . Interplay between the cancer genome and epigenome. Cell. 2013;153:38–55. 23540689 10.1016/j.cell.2013.03.008 PMC3648790

[6] Haffner MC , Chaux A , Meeker AK , et al. Global 5‐hydroxymethylcytosine content is significantly reduced in tissue stem/progenitor cell compartments and in human cancers. Oncotarget. 2011;2:627–637. 21896958 10.18632/oncotarget.316 PMC3248214

[7] Yang H , Liu Y , Bai F , et al. Tumor development is associated with decrease of TET gene expression and 5‐methylcytosine hydroxylation. Oncogene. 2013;32:663–669. 22391558 10.1038/onc.2012.67 PMC3897214

[8] Gupta SM , Mania‐Pramanik J . Molecular mechanisms in progression of HPV‐associated cervical carcinogenesis. J Biomed Sci. 2019;26:28. 31014351 10.1186/s12929-019-0520-2 PMC6477741

[9] Verlaat W , Van Leeuwen RW , Novianti PW , et al. Host‐cell DNA methylation patterns during high‐risk HPV‐induced carcinogenesis reveal a heterogeneous nature of cervical pre‐cancer. Epigenetics. 2018;13:769–778. 30079796 10.1080/15592294.2018.1507197 PMC6224221

[10] Wang J , Su Y , Tian Y , et al. Characterization of DNA hydroxymethylation profile in cervical cancer. Artif Cells Nanomed Biotechnol. 2019;47:2706–2714. 31271297 10.1080/21691401.2019.1634578

[11] Sun Z , Asmann YW , Kalari KR , et al. Integrated analysis of gene expression, CpG island methylation, and gene copy number in breast cancer cells by deep sequencing. PLoS One. 2011;6:e17490. 21364760 10.1371/journal.pone.0017490 PMC3045451

[12] Miyashita N , Horie M , Suzuki HI , et al. An integrative analysis of transcriptome and epigenome features of ASCL1‐positive lung adenocarcinomas. J Thorac Oncol. 2018;13:1676–1691. 30121393 10.1016/j.jtho.2018.07.096

[13] Zheng Y , Huang Q , Ding Z , et al. Genome‐wide DNA methylation analysis identifies candidate epigenetic markers and drivers of hepatocellular carcinoma. Brief Bioinform. 2018;19:101–108. 27760737 10.1093/bib/bbw094

[14] Li Q , Wang P , Sun C , et al. Integrative analysis of methylation and transcriptome identified epigenetically regulated lncRNAs with prognostic relevance for thyroid cancer. Front Bioeng Biotechnol. 2019;7:439. 31998704 10.3389/fbioe.2019.00439 PMC6962111

[15] Jin Y , Qin X . Integrated analysis of DNA methylation and mRNA expression profiles to identify key genes in head and neck squamous cell carcinoma. Biosci Rep. 2020;40 :BSR20193349. 31894857 10.1042/BSR20193349 PMC6981101

[16] Liu L , He D , Wang Y , et al. Integrated analysis of DNA methylation and transcriptome profiling of polycystic ovary syndrome. Mol Med Rep. 2020;21:2138–2150. 32323770 10.3892/mmr.2020.11005 PMC7115196

[17] Münzel M , Globisch D , Carell T . 5‐Hydroxymethylcytosine, the sixth base of the genome. Angew Chem Int Ed Engl. 2011;50:6460–6468. 21688365 10.1002/anie.201101547

[18] Bachman M , Uribe‐Lewis S , Yang X , et al. 5‐Hydroxymethylcytosine is a predominantly stable DNA modification. Nat Chem. 2014;6:1049–1055. 25411882 10.1038/nchem.2064 PMC4382525

[19] Lian Z‐Q , Wang Q , Li W‐P , et al. Screening of significantly hypermethylated genes in breast cancer using microarray‐based methylated‐CpG island recovery assay and identification of their expression levels. Int J Oncol. 2012;41:629–638. 22581028 10.3892/ijo.2012.1464

[20] Schmitt M , Wilhelm OG , Noske A , et al. Clinical validation of DNA methylation to predict outcome in high‐risk breast cancer patients treated with anthracycline‐based chemotherapy. Breast Care (Basel). 2018;13:425–433. 30800037 10.1159/000493016 PMC6381917

[21] Miotto E , Sabbioni S , Veronese A , et al. Frequent aberrant methylation of the CDH4 gene promoter in human colorectal and gastric cancer. Cancer Res. 2004;64:8156–8159. 15548679 10.1158/0008-5472.CAN-04-3000

[22] Böttcher R , Dulla K , van Strijp D , et al. Human PDE4D isoform composition is deregulated in primary prostate cancer and indicative for disease progression and development of distant metastases. Oncotarget. 2016;7:70669–70684. 27683107 10.18632/oncotarget.12204 PMC5342582

[23] Sepulveda JL , Gutierrez‐Pajares JL , Luna A , et al. High‐definition CpG methylation of novel genes in gastric carcinogenesis identified by next‐generation sequencing. Mod Pathol. 2016;29:182–193. 26769141 10.1038/modpathol.2015.144

[24] Guo H , Zhu P , Yan L , et al. The DNA methylation landscape of human early embryos. Nature. 2014;511:606–610. 25079557 10.1038/nature13544

[25] Huang J , Qian Z , Gong Y , et al. Comprehensive genomic variation profiling of cervical intraepithelial neoplasia and cervical cancer identifies potential targets for cervical cancer early warning. J Med Genet. 2019;56:186–194. 30567904 10.1136/jmedgenet-2018-105745 PMC6581088

[26] Chan KC , Jiang P , Chan CW , et al. Noninvasive detection of cancer‐associated genome‐wide hypomethylation and copy number aberrations by plasma DNA bisulfite sequencing. Proc Natl Acad Sci U S A. 2013;110:18761–18768. 24191000 10.1073/pnas.1313995110 PMC3839703

[27] Roadmap Epigenomics C , Kundaje A , Meuleman W , et al. Integrative analysis of 111 reference human epigenomes. Nature. 2015;518:317–330. 25693563 10.1038/nature14248 PMC4530010

[28] Pfister SX , Ahrabi S , Zalmas LP , et al. SETD2‐dependent histone H3K36 trimethylation is required for homologous recombination repair and genome stability. Cell Rep. 2014;7:2006–2018. 24931610 10.1016/j.celrep.2014.05.026 PMC4074340

[29] Chen K , Zhang J , Guo Z , et al. Loss of 5‐hydroxymethylcytosine is linked to gene body hypermethylation in kidney cancer. Cell Res. 2016;26:103–118. 26680004 10.1038/cr.2015.150 PMC4816137

[30] Teschendorff AE , Gao Y , Jones A , et al. DNA methylation outliers in normal breast tissue identify field defects that are enriched in cancer. Nat Commun. 2016;7:10478. 26823093 10.1038/ncomms10478 PMC4740178

[31] Teschendorff AE , Jones A , Fiegl H , et al. Epigenetic variability in cells of normal cytology is associated with the risk of future morphological transformation. Genome Medicine. 2012;4:24. 22453031 10.1186/gm323 PMC3446274

[32] Esteller M . Epigenetics in cancer. N Engl J Med. 2008;358:1148–1159. 18337604 10.1056/NEJMra072067

[33] Teschendorff AE , Liu X , Caren H , et al. The dynamics of DNA methylation covariation patterns in carcinogenesis. PLoS Comput Biol. 2014;10:e1003709. 25010556 10.1371/journal.pcbi.1003709 PMC4091688

[34] Huang KK , Ramnarayanan K , Zhu F , et al. Genomic and epigenomic profiling of high‐risk intestinal metaplasia reveals molecular determinants of progression to gastric cancer. Cancer Cell. 2018;33:137–150.e5 29290541 10.1016/j.ccell.2017.11.018

[35] Jiao X , Zhang S , Jiao J , et al. Promoter methylation of SEPT9 as a potential biomarker for early detection of cervical cancer and its overexpression predicts radioresistance. Clin Epigenetics. 2019;11:120. 31426855 10.1186/s13148-019-0719-9 PMC6700799

[36] De Strooper LMA , Berkhof J , Steenbergen RDM , et al. Cervical cancer risk in HPV‐positive women after a negative FAM19A4/mir124‐2 methylation test: a post hoc analysis in the POBASCAM trial with 14 year follow‐up. Int J Cancer. 2018;143:1541–1548. 29663363 10.1002/ijc.31539 PMC6099282

[37] Wijetunga NA , Belbin TJ , Burk RD , et al. Novel epigenetic changes in CDKN2A are associated with progression of cervical intraepithelial neoplasia. Gynecol Oncol. 2016;142:566–573. 27401842 10.1016/j.ygyno.2016.07.006 PMC5125392

[38] Li T , Fan J , Wang B , et al. TIMER: a web server for comprehensive analysis of tumor‐infiltrating immune cells. Cancer Res. 2017;77:e108–e110. 29092952 10.1158/0008-5472.CAN-17-0307 PMC6042652

[39] Hopman ANH , Moshi JM , Hoogduin KJ , et al. SOX17 expression and its down‐regulation by promoter methylation in cervical adenocarcinoma in situ and adenocarcinoma. Histopathology. 2020;76:383–393. 31444787 10.1111/his.13980 PMC7027543

[40] Qin R , Cao L , Wang J , et al. Promoter methylation of ezrin and its impact on the incidence and prognosis of cervical cancer. Cell Physiol Biochem. 2018;50:277–287. 30282070 10.1159/000494005

[41] Su PH , Lai HC , Huang RL , et al. Paired box‐1 (PAX1) activates multiple phosphatases and inhibits kinase cascades in cervical cancer. Sci Rep. 2019;9:9195. 31235851 10.1038/s41598-019-45477-5 PMC6591413

[42] Li X , Wu X , Li Y , et al. Promoter hypermethylation of SOX11 promotes the progression of cervical cancer in vitro and in vivo. Oncol Rep. 2019;41:2351–2360. 30720133 10.3892/or.2019.6993

[43] Rogeri CD , Silveira HCS , Causin RL , et al. Methylation of the hsa‐miR‐124, SOX1, TERT, and LMX1A genes as biomarkers for precursor lesions in cervical cancer. Gynecol Oncol. 2018;150:545–551. 29960712 10.1016/j.ygyno.2018.06.014

[44] Shu R , He J , Wu C , et al. The association between RARbeta and FHIT promoter methylation and the carcinogenesis of patients with cervical carcinoma: a meta‐analysis. Tumour Biol. 2017;39:1010428317709126. 28639889 10.1177/1010428317709126

[45] Kocsis A , Takacs T , Jeney C , et al. Performance of a new HPV and biomarker assay in the management of hrHPV positive women: subanalysis of the ongoing multicenter TRACE clinical trial (n >6,000) to evaluate POU4F3 methylation as a potential biomarker of cervical precancer and cancer. Int J Cancer. 2017;140:1119–1133. 27874187 10.1002/ijc.30534

[46] Shen‐Gunther J , Wang CM , Poage GM , et al. Molecular Pap smear: hPV genotype and DNA methylation of ADCY8, CDH8, and ZNF582 as an integrated biomarker for high‐grade cervical cytology. Clin Epigenetics. 2016;8:96. 27651839 10.1186/s13148-016-0263-9 PMC5022163

[47] Sood S , Patel FD , Ghosh S , et al. Epigenetic alteration by DNA methylation of ESR1, MYOD1 and hTERT gene promoters is useful for prediction of response in patients of locally advanced invasive cervical carcinoma treated by chemoradiation. Clin Oncol (R Coll Radiol). 2015;27:720–727. 26344356 10.1016/j.clon.2015.08.001

[48] Thangavelu PU , Krenacs T , Dray E , et al. In epithelial cancers, aberrant COL17A1 promoter methylation predicts its misexpression and increased invasion. Clin Epigenetics. 2016;8:120. 27891193 10.1186/s13148-016-0290-6 PMC5116176

[49] Yin A , Zhang Q , Kong X , et al. JAM3 methylation status as a biomarker for diagnosis of preneoplastic and neoplastic lesions of the cervix. Oncotarget. 2015;6:44373–44387. 26517242 10.18632/oncotarget.6250 PMC4792563

[50] Verlaat W , Snoek BC , Heideman DAM , et al. Identification and validation of a 3‐gene methylation classifier for HPV‐based cervical screening on self‐samples. Clin Cancer Res. 2018;24:3456–3464. 29632006 10.1158/1078-0432.CCR-17-3615 PMC6053041

[51] Taryma‐Lesniak O , Sokolowska KE , Wojdacz TK . Current status of development of methylation biomarkers for in vitro diagnostic IVD applications. Clin Epigenetics. 2020;12:100. 32631437 10.1186/s13148-020-00886-6 PMC7336678

[52] McLaughlin‐Drubin ME , Crum CP , Munger K . Human papillomavirus E7 oncoprotein induces KDM6A and KDM6B histone demethylase expression and causes epigenetic reprogramming. Proc Natl Acad Sci U S A. 2011;108:2130–2135. 21245294 10.1073/pnas.1009933108 PMC3033314

[53] Bo H , Fan L , Gong Z , et al. Upregulation and hypomethylation of lncRNA AFAP1AS1 predicts a poor prognosis and promotes the migration and invasion of cervical cancer. Oncol Rep. 2019;41:2431–2439. 30816545 10.3892/or.2019.7027

[54] Zhang J , Lin Z , Gao Y , et al. Downregulation of long noncoding RNA MEG3 is associated with poor prognosis and promoter hypermethylation in cervical cancer. J Exp Clin Cancer Res. 2017;36:5. 28057015 10.1186/s13046-016-0472-2 PMC5216566

[55] Zummeren MV , Kremer WW , Leeman A , et al. HPV E4 expression and DNA hypermethylation of CADM1, MAL, and miR124‐2 genes in cervical cancer and precursor lesions. Mod Pathol. 2018;31:1842–1850. 30135508 10.1038/s41379-018-0101-z

[56] Panagopoulou M , Lambropoulou M , Balgkouranidou I , et al. Gene promoter methylation and protein expression of BRMS1 in uterine cervix in relation to high‐risk human papilloma virus infection and cancer. Tumour Biol. 2017;39:1010428317697557. 28381193 10.1177/1010428317697557

[57] Rieke DT , Ochsenreither S , Klinghammer K , et al. Methylation of RAD51B, XRCC3 and other homologous recombination genes is associated with expression of immune checkpoints and an inflammatory signature in squamous cell carcinoma of the head and neck, lung and cervix. Oncotarget. 2016;7:75379‐75393. 27683114 10.18632/oncotarget.12211 PMC5342748

[58] He C , Mao D , Hua G , et al. The Hippo/YAP pathway interacts with EGFR signaling and HPV oncoproteins to regulate cervical cancer progression. EMBO Mol Med. 2015;7:1426–1449. 26417066 10.15252/emmm.201404976 PMC4644376

[59] Buglioni S , Vici P , Sergi D , et al. Analysis of the hippo transducers TAZ and YAP in cervical cancer and its microenvironment. Oncoimmunology. 2016;5:e1160187. 27471633 10.1080/2162402X.2016.1160187 PMC4938371

[60] Yang S , Wu Y , Wang S , et al. HPV‐related methylation‐based reclassification and risk stratification of cervical cancer. Mol Oncol. 2020;14:2124–2141. 32408396 10.1002/1878-0261.12709 PMC7463306

[61] Wu X , Peng L , Zhang Y , et al. Identification of key genes and pathways in cervical cancer by bioinformatics analysis. Int J Med Sci. 2019;16:800–812. 31337953 10.7150/ijms.34172 PMC6643108

[62] Jin Z , Wang L , Zhang Y , et al. MAL hypermethylation is a tissue‐specific event that correlates with MAL mRNA expression in esophageal carcinoma. Sci Rep. 2013;3:2838. 24088706 10.1038/srep02838 PMC3789153

[63] Semenas J , Hedblom A , Miftakhova RR , et al. The role of PI3K/AKT‐related PIP5K1α and the discovery of its selective inhibitor for treatment of advanced prostate cancer. Proc Natl Acad Sci U S A. 2014;111:E3689–E3698. 25071204 10.1073/pnas.1405801111 PMC4156761

[64] Drake JM , Huang J . PIP5K1α inhibition as a therapeutic strategy for prostate cancer. Proc Natl Acad Sci U S A. 2014;111:12578–12579. 25118275 10.1073/pnas.1413363111 PMC4156720

[65] Dewdney SB , Rimel BJ , Thaker PH , et al. Aberrant methylation of the X‐linked ribosomal S6 kinase RPS6KA6 (RSK4) in endometrial cancers. Clin Cancer Res. 2011;17:2120–2129. 21372219 10.1158/1078-0432.CCR-10-2668 PMC3242504

[66] Huo H , Ye X , Yang H , et al. RSK4 inhibits breast cancer cell proliferation and invasion in vitro, and is correlated with estrogen receptor upregulation in breast cancer. Oncol Rep. 2019;42:2777–2787. 31545499 10.3892/or.2019.7328

[67] Fan L , Li P , Yin Z , et al. Ribosomal s6 protein kinase 4: a prognostic factor for renal cell carcinoma. Br J Cancer. 2013;109:1137–1146. 23942078 10.1038/bjc.2013.463 PMC3778307

[68] Li M‐Y , Fan L‐N , Han D‐H , et al. Ribosomal S6 protein kinase 4 promotes radioresistance in esophageal squamous cell carcinoma. J Clin Invest. 2020;130:4301–4319. 32396532 10.1172/JCI134930 PMC7410060

[69] Zhang Y , Zou X , Qian W , et al. Enhanced PAPSS2/VCAN sulfation axis is essential for Snail‐mediated breast cancer cell migration and metastasis. Cell Death Differ. 2019;26:565–579. 29955124 10.1038/s41418-018-0147-y PMC6370781

[70] Pinto PH , Kroupova A , Schleiffer A , et al. ANGEL2 is a member of the CCR4 family of deadenylases with 2',3'‐cyclic phosphatase activity. Science. 2020;369:524–530. 32732418 10.1126/science.aba9763

[71] Pitre A , Ge Y , Lin W , et al. An unexpected protein interaction promotes drug resistance in leukemia. Nat Commun. 2017;8:1547. 29146910 10.1038/s41467-017-01678-y PMC5691054

[72] Supek F , Lehner B , Hajkova P , et al. Hydroxymethylated cytosines are associated with elevated C to G transversion rates. PLoS Genet. 2014;10:e1004585. 25211471 10.1371/journal.pgen.1004585 PMC4161303

[73] Tomkova M , McClellan M , Kriaucionis S , et al. 5‐hydroxymethylcytosine marks regions with reduced mutation frequency in human DNA. Elife. 2016;5:e17082. 27183007 10.7554/eLife.17082 PMC4931910

[74] Feber A , Guilhamon P , Lechner M , et al. Using high‐density DNA methylation arrays to profile copy number alterations. Genome Biol. 2014;15:R30. 24490765 10.1186/gb-2014-15-2-r30 PMC4054098

[75] Soto D , Song C , McLaughlin‐Drubin ME . Epigenetic alterations in human papillomavirus‐associated cancers. Viruses. 2017;9:248. 10.3390/v9090248 PMC5618014 28862667

[76] Dalla Palma P , Giorgi Rossi P , Collina G , et al. The reproducibility of CIN diagnoses among different pathologists: data from histology reviews from a multicenter randomized study. Am J Clin Pathol. 2009;132:125–132. 19864243 10.1309/AJCPBRK7D1YIUWFP

[77] Xi Y , Li W . BSMAP: whole genome bisulfite sequence MAPping program. BMC Bioinformatics. 2009;10:232. 19635165 10.1186/1471-2105-10-232 PMC2724425

[78] Qu J , Zhou M , Song Q , et al. MLML: consistent simultaneous estimates of DNA methylation and hydroxymethylation. Bioinformatics. 2013;29:2645–2646. 23969133 10.1093/bioinformatics/btt459 PMC3789553

[79] Jühling F , Kretzmer H , Bernhart SH , et al. metilene: fast and sensitive calling of differentially methylated regions from bisulfite sequencing data. Genome Res. 2016;26:256–262. 26631489 10.1101/gr.196394.115 PMC4728377

[80] Ziller MJ , Hansen KD , Meissner A , et al. Coverage recommendations for methylation analysis by whole‐genome bisulfite sequencing. Nat Methods. 2015;12:230–232. 25362363 10.1038/nmeth.3152 PMC4344394

[81] Li X , Liu Y , Salz T , et al. Whole‐genome analysis of the methylome and hydroxymethylome in normal and malignant lung and liver. Genome Res. 2016;26:1730–1741. 27737935 10.1101/gr.211854.116 PMC5131824

[82] Liu Y , Siegmund KD , Laird PW , et al. Bis‐SNP: combined DNA methylation and SNP calling for Bisulfite‐seq data. Genome Biol. 2012;13:R61. 22784381 10.1186/gb-2012-13-7-r61 PMC3491382

[83] Amarasinghe KC , Li J , Hunter SM , et al. Inferring copy number and genotype in tumour exome data. BMC Genomics. 2014;15:732. 25167919 10.1186/1471-2164-15-732 PMC4162913

[84] Miller CA , Hampton O , Coarfa C , et al. ReadDepth: a parallel R package for detecting copy number alterations from short sequencing reads. PLoS One. 2011;6:e16327. 21305028 10.1371/journal.pone.0016327 PMC3031566

[85] Yu G , Wang L‐G , Han Y , et al. clusterProfiler: an R package for comparing biological themes among gene clusters. OMICS. 2012;16:284–287. 22455463 10.1089/omi.2011.0118 PMC3339379

[86] Andersson R , Gebhard C , Miguel‐Escalada I , et al. An atlas of active enhancers across human cell types and tissues. Nature. 2014;507:455–461. 24670763 10.1038/nature12787 PMC5215096

[87] Cavalcante RG , Sartor MA . annotatr: genomic regions in context. Bioinformatics. 2017;33:2381–2383. 28369316 10.1093/bioinformatics/btx183 PMC5860117

