# Supplementary material for: An epigenomic landscape of cervical intraepithelial neoplasia and cervical cancer using single‐base resolution methylome and hydroxymethylome
Source: Clin Transl Med. 2021 Jul 19;11(7):e498. doi: 10.1002/ctm2.498 (PMC8288011; doi:10.1002/ctm2.498)
Supplement: Supplementary file 1 — figureS1‐S8 [file CTM2-11-e498-s006.docx]

**Appendices**

**Supplementary Figures**

**The file includes:**

Fig. S1-S8

**
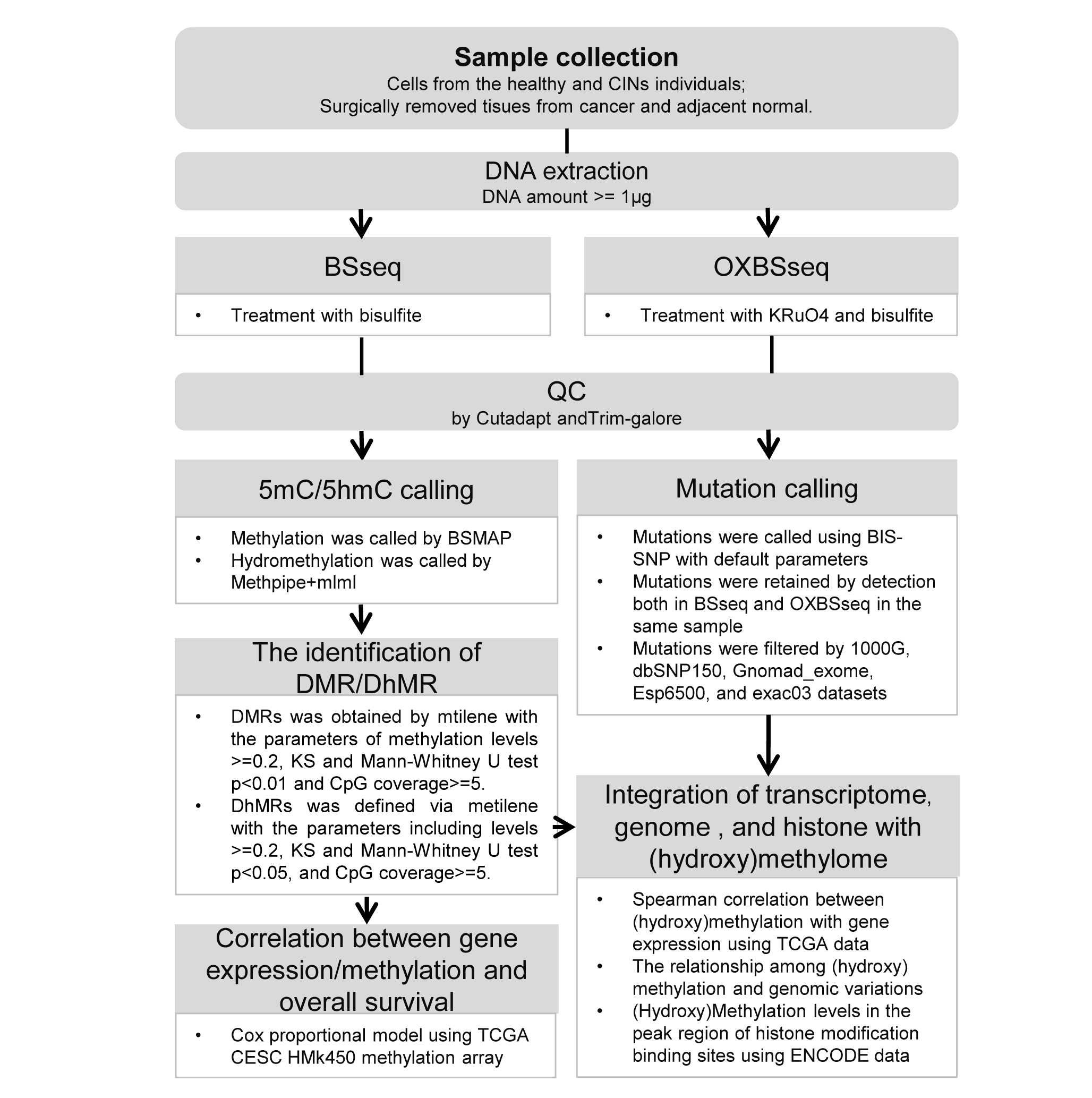
**

**Supplementary FIGURE S1.** Bioinformatical Flow Chart.

**
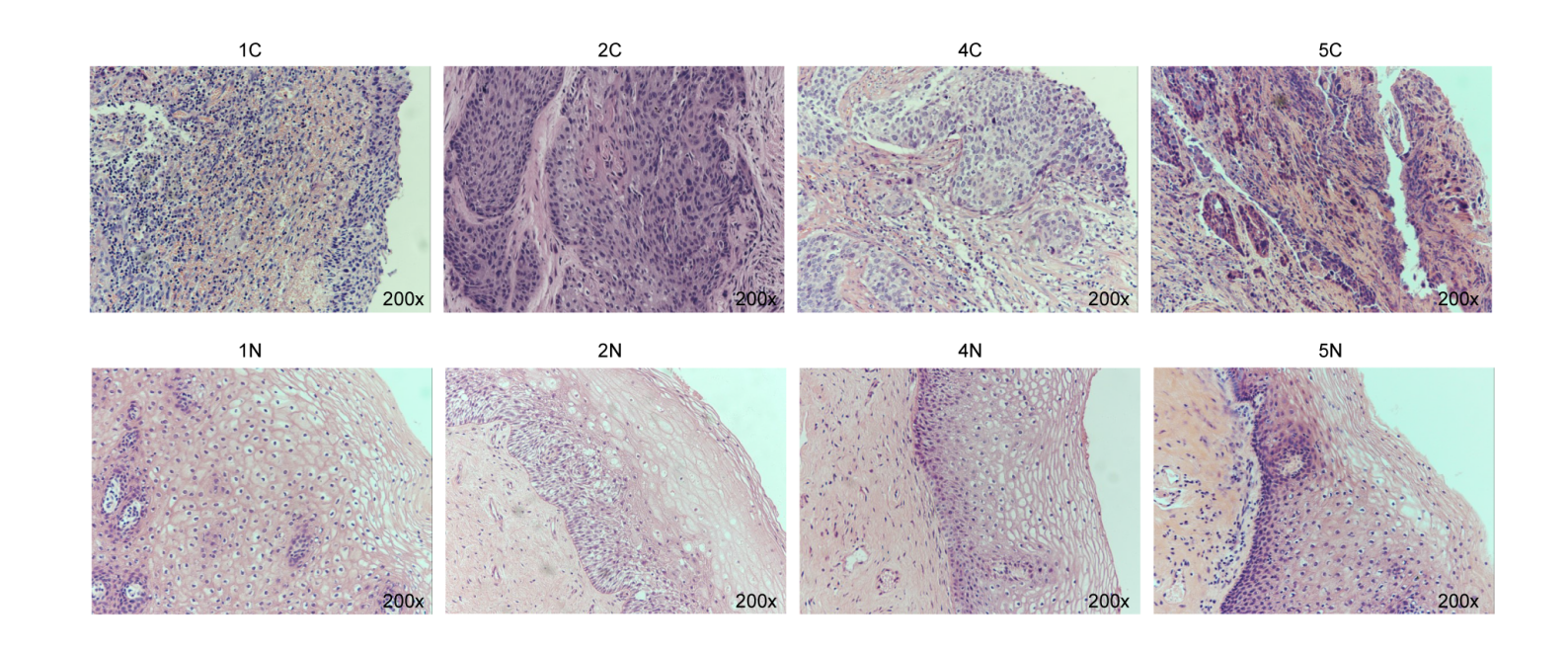
**

**Supplementary FIGURE S2.** The images showing the H&E staining of tumor-normal paired tissues.

**
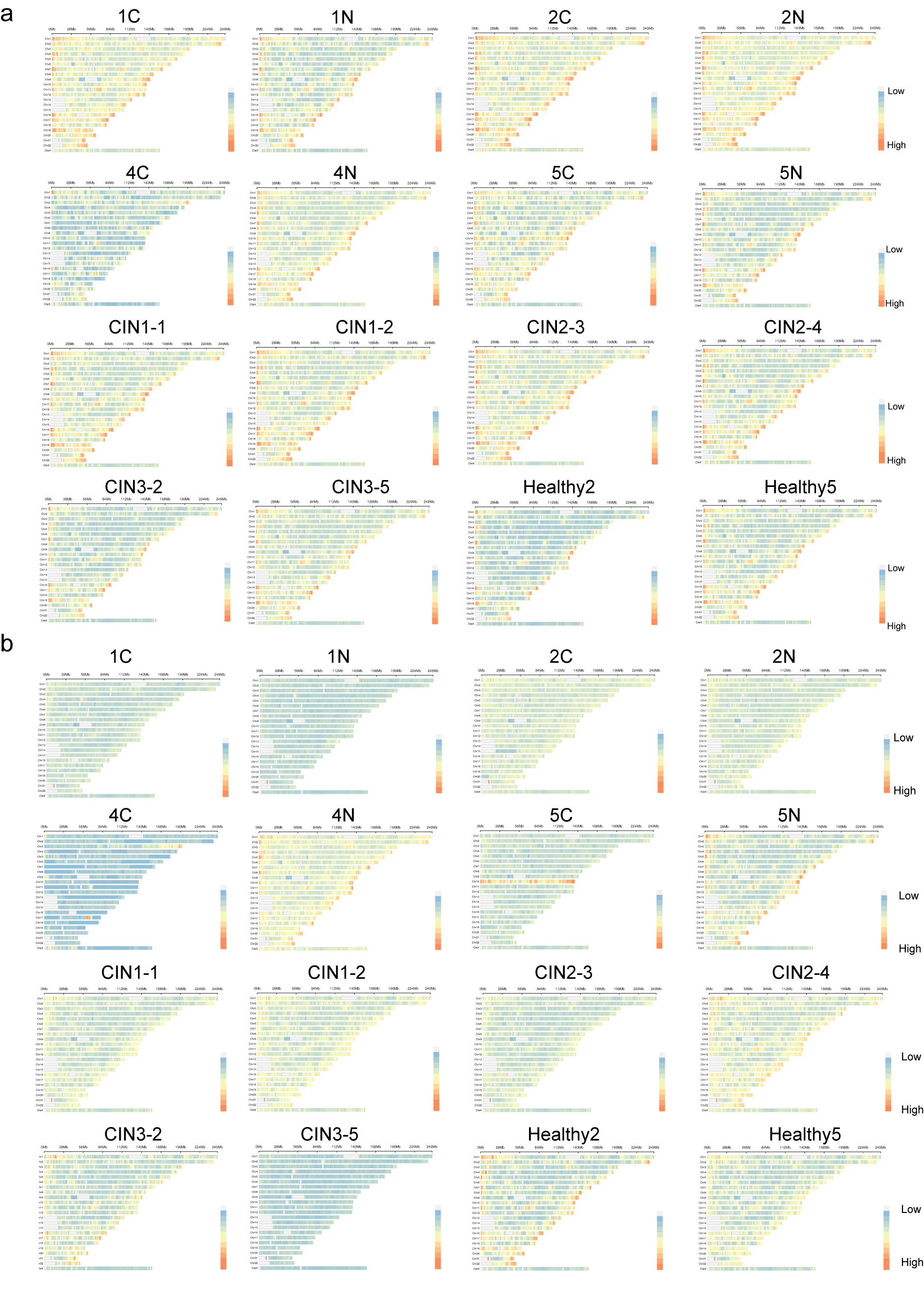
**

**Supplementary FIGURE S3.** Chromosome distribution of covered methylated (a) and hydroxymethylated (b) intergenic CpGs. C: Cervical cancer. N: adjacent paracancer tissue.


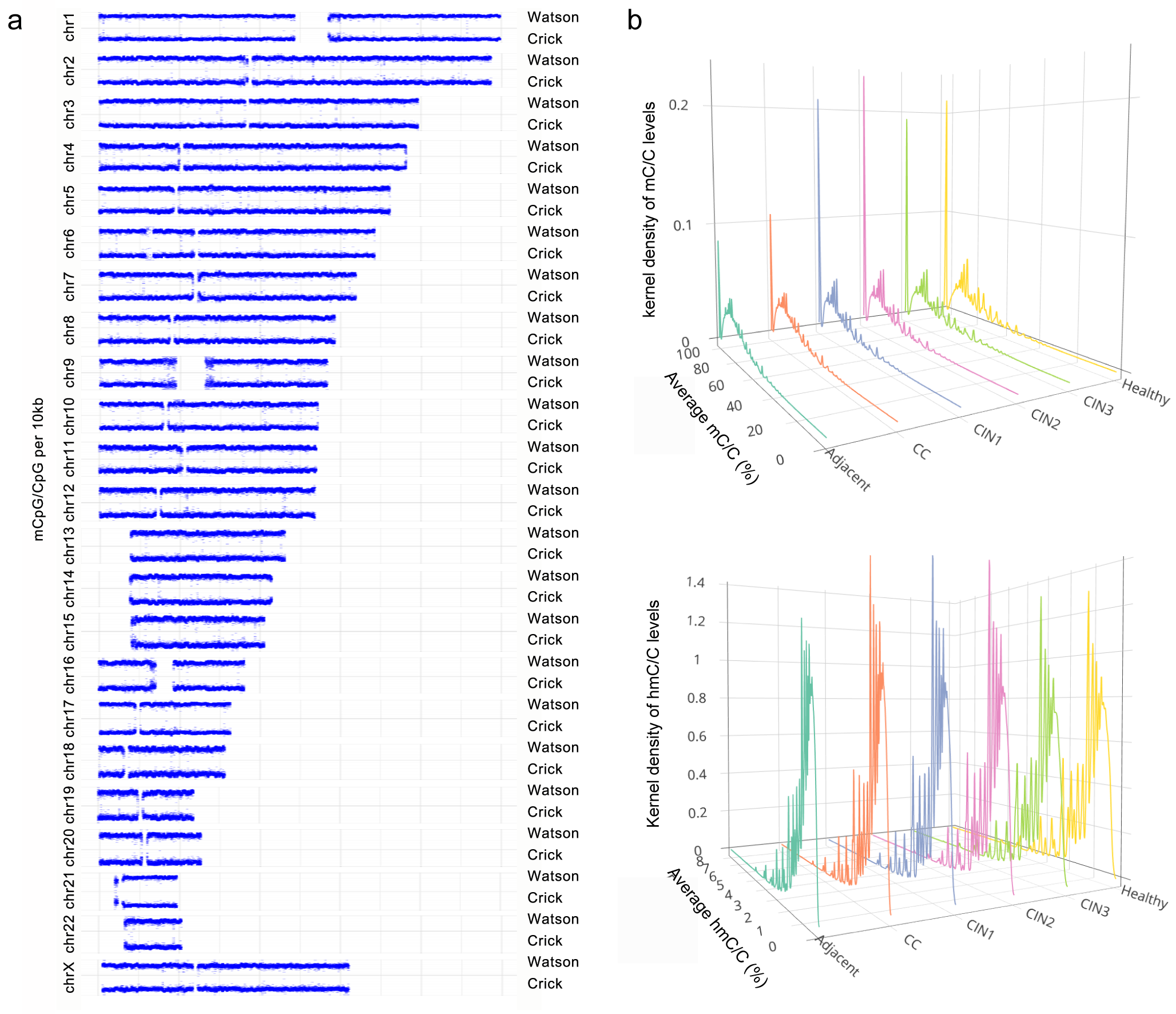


**Supplementary FIGURE S4.** (a) The methylation level in watson and crick strand across 23 chromosomes. (b) Density plot of methylation levels (upper) and hydroxymethylation levels (lower) in the Healthy, CINs, CC, and adjacent tissues.


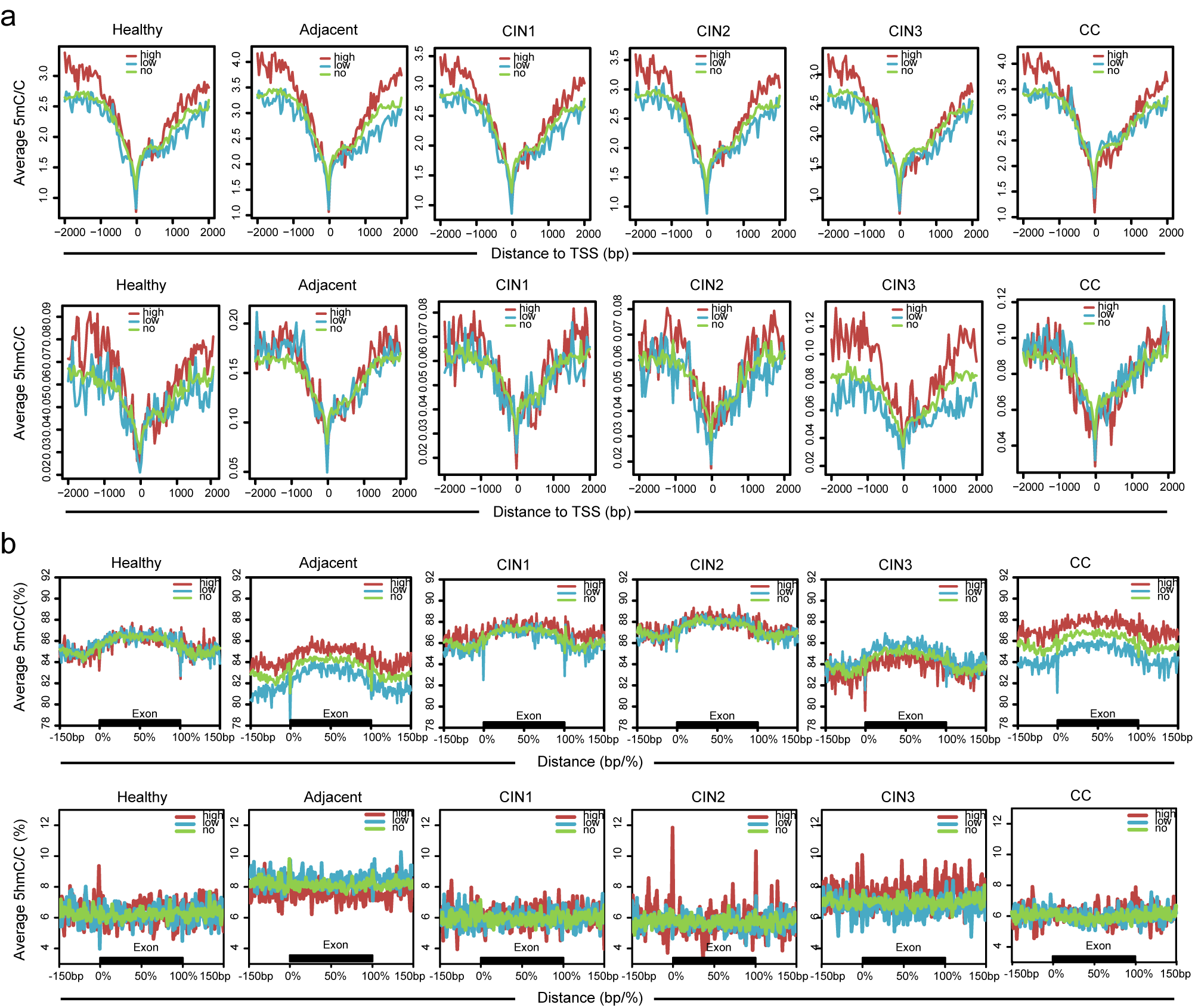


**Supplementary FIGURE S5.** RNA differentially expressed and the level of methylation (a)/ hydroxymethylation (b) at TSS regions and 2kb flanking region under different states (high, low, or neutral).

**
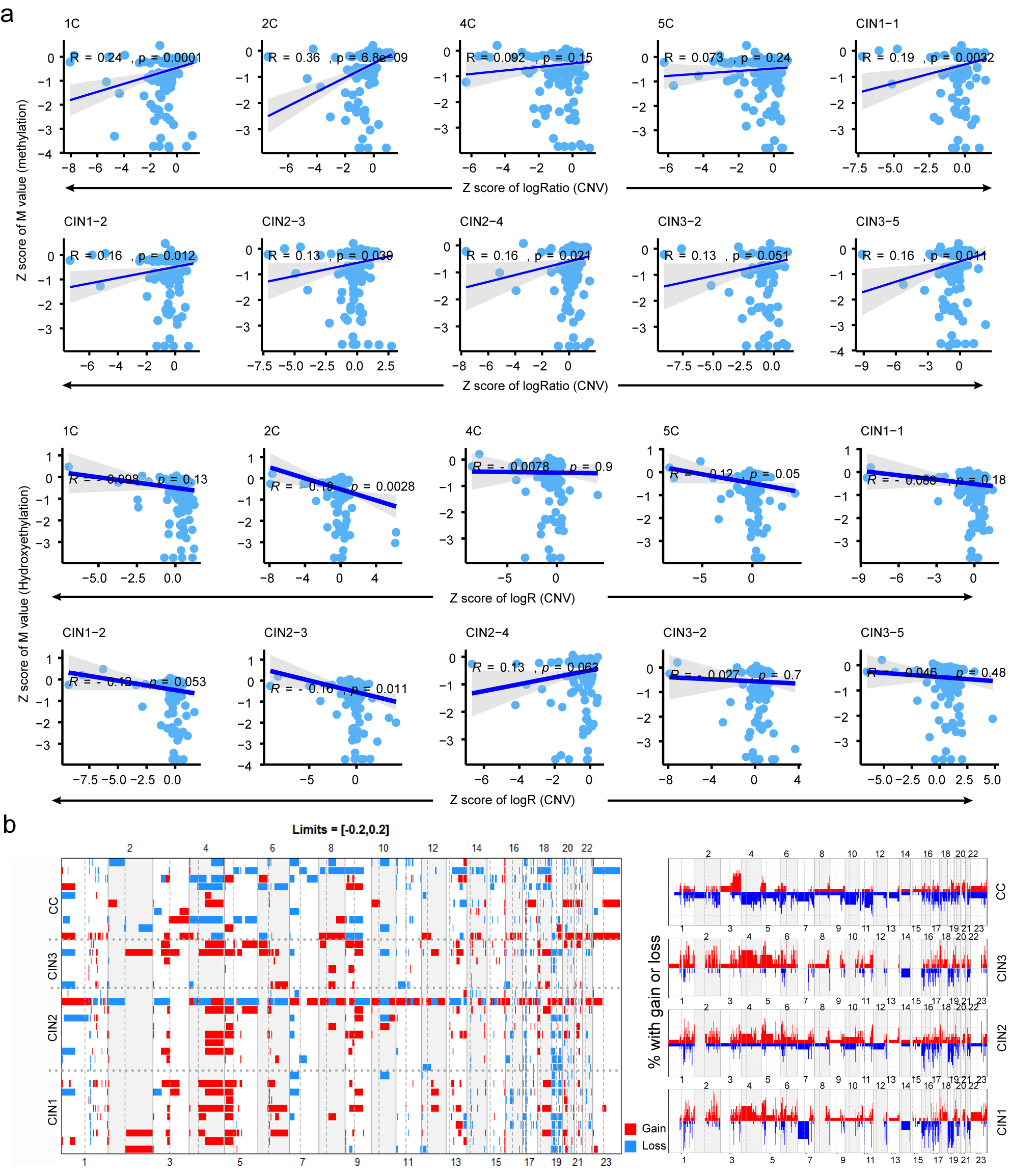
**

**Supplementary FIGURE S6.** The SCNV changes in the progression to cervical carcinogenesis and the correlation with methylation/hydroxymethylation. (a) The pair-wise spearman correlation of CNV and methylation (upper panel) or hydroxymethylation (lower panel) for each sample using bisulfite sequencing data. (b) The heatmap (left panel; red, gain; blue, loss) and chrom-plot (right panel) depicting the CNV alterations in CINs and CC using whole exome sequencing data.

**
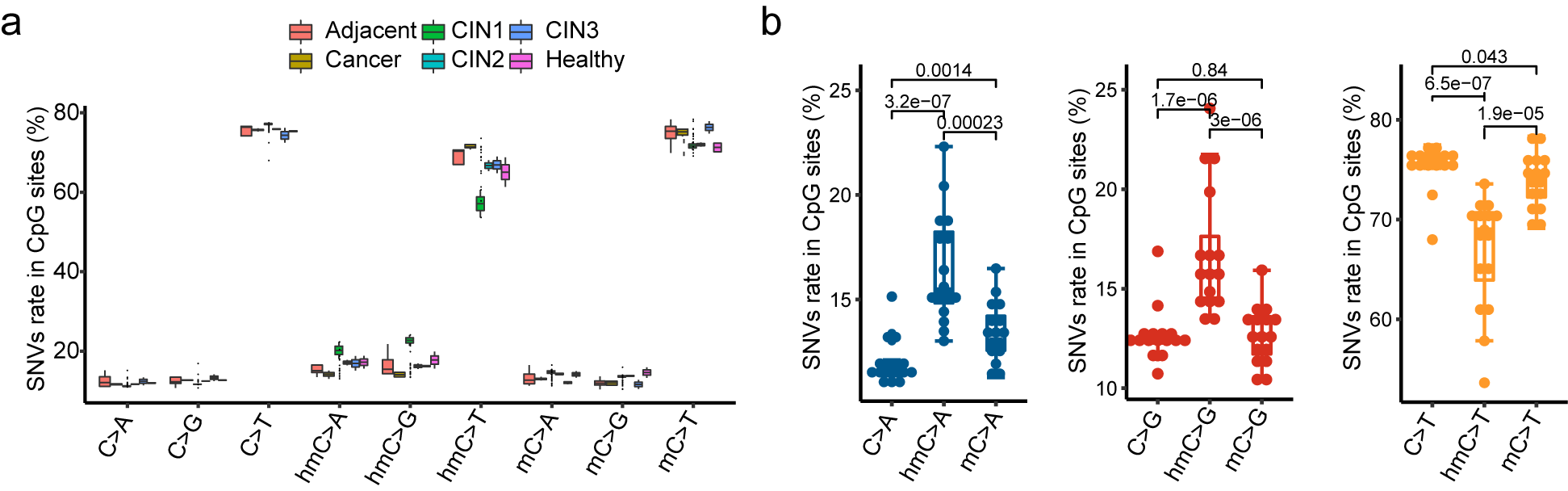
**

**Supplementary FIGURE S7.** Substitution rates of cytosines in CpG sites. (a) The SNVs mutation rates in CpG sites with or without cytosine methylation modification in the healthy, CINs, CC and adjacent paracancer tissues. (b) The overall SNVs mutation rates of C>A, C>G, C>T with or without cytosine methylation in CpG sites.


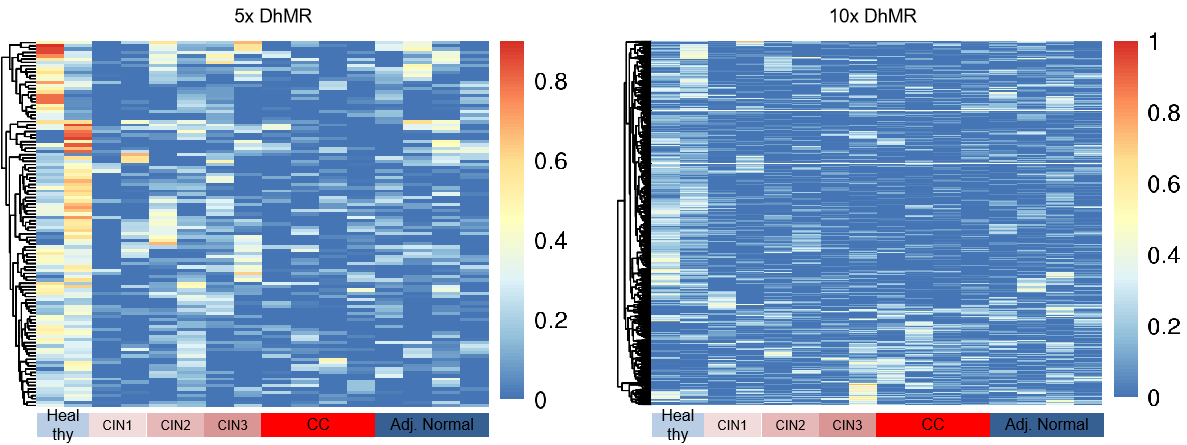


**Supplementary FIGURE S8.** The DhMR in the healthy, CINs and CC detected by metilene with different thresholds. The heatmaps depicting the beta values of each CpGs within DhMR filtered by (left) 5x coverage, or (right) 10x coverage.
